# Changes in DNA Methylation from Age 18 to Pregnancy in Type 1, 2, and 17 T Helper and Regulatory T-Cells Pathway Genes

**DOI:** 10.3390/ijms19020477

**Published:** 2018-02-06

**Authors:** Sabrina Iqbal, Gabrielle A. Lockett, John W. Holloway, S. Hasan Arshad, Hongmei Zhang, Akhilesh Kaushal, Sabarinath R. Tetali, Nandini Mukherjee, Wilfried J. J. Karmaus

**Affiliations:** 1 Division of Epidemiology, Biostatistics and Environmental Health, School of Public Health, University of Memphis, Memphis, 301 Robison Hall, 3825 DeSoto Avenue Memphis, TN 38152, USA; sabrina1.iqbal@gmail.com (S.I.); hzhang6@memphis.edu (H.Z.); akaushl1@memphis.edu (A.K.); sabarinath5889@gmail.com (S.R.T.); nmkhrjee@memphis.edu (N.M.); 2 Human Development and Health, Faculty of Medicine, University of Southampton, Southampton SO17 1BJ, UK; G.A.Lockett@soton.ac.uk (G.A.L.); J.W.Holloway@soton.ac.uk (J.W.H.); 3 Faculty of Medicine, University of Southampton, Southampton SO17 1BJ, UK; S.H.Arshad@soton.ac.uk; 4 The David Hide Asthma and Allergy Research Centre, Newport PO30 5TG, UK

**Keywords:** Epigenetics, genes, DNA methylation, pregnancy, Th1 bias, Th2 bias, Th17 bias, regulatory T cells

## Abstract

To succeed, pregnancies need to initiate immune biases towards T helper 2 (Th2) responses, yet little is known about what establishes this bias. Using the Illumina 450 K platform, we explored changes in DNA methylation (DNAm) of Th1, Th2, Th17, and regulatory T cell pathway genes before and during pregnancy. Female participants were recruited at birth (1989), and followed through age 18 years and their pregnancy (2011–2015). Peripheral blood DNAm was measured in 245 girls at 18 years; from among these girls, the DNAm of 54 women was repeatedly measured in the first (weeks 8–21, *n* = 39) and second (weeks 22–38, *n* = 35) halves of pregnancy, respectively. M-values (logit-transformed β-values of DNAm) were analyzed: First, with repeated measurement models, cytosine–phosphate–guanine sites (CpGs) of pathway genes in pregnancy and at age 18 (nonpregnant) were compared for changes (*p* ≤ 0.05). Second, we tested how many of the 348 pathway-related CpGs changed compared to 10 randomly selected subsets of all other CpGs and compared to 10 randomly selected subsets of other CD4+-related CpGs (348 in each subset). Contrasted to the nonpregnant state, 27.7% of Th1-related CpGs changed in the first and 36.1% in the second half of pregnancy. Among the Th2 pathway CpGs, proportions of changes were 35.1% (first) and 33.8% (second half). The methylation changes suggest involvement of both Th1 and Th2 pathway CpGs in the immune bias during pregnancy. Changes in regulatory T cell and Th17 pathways need further exploration.

## 1. Introduction

Pregnancy initiates a unique state of immune changes including a bias towards T helper 2 (Th2) responses, observed in several studies [1,2,3,4]. However, there is insufficient knowledge regarding mechanisms that drive these changes, and whether DNA methylation (DNAm) is involved in this process. The gestational immune system plays an important role in defending both mother and fetus against foreign antigens [1,5]. Immune cells at the implantation site help to protect the pregnancy by modifying the response tolerating the presence of a fetus [6]. During pregnancy, the fetal and maternal immune systems show a co-acting state; supporting one another [7]. An insufficient maternal immune deviation during pregnancy is considered to be related to adverse pregnancy outcomes such as abortions (miscarriages) [4,5].

Immune responses have been divided into two patterns—the innate and the adaptive immune system [1,8]. The innate immune responses provide an immediate defense against pathogens, whereas the adaptive immune system reacts to specific antigens [9]. Adaptive immune responses are mediated by T and B lymphocytes. B cells and their antibodies initiate humoral immunity; T cells primarily provide cell-mediated immunity [8,10]. T cells represent a subset of T helper (CD4+) cells which have been subdivided into Th1, Th2, Th17, and regulatory T cells (Treg). The Th1 response is characterized by pro-inflammatory cytokines, whereas the Th2 response is anti-inflammatory [1,8]. The Th17 pathway plays an important role in inducing inflammation [11,12]; Treg cells inhibit inflammation and are responsible for immune regulation.

Th1 cells produce proteins encoded by genes of the *IFN-γ* family [12,13,14,15,16,17,18,19,20,21,22,23], *TNF-α* family [12,13,14,15,16,17,18,19,20,21,22,23], *IL-2* [12,13,14,17,20,22,23], and the *IL-12* family [13,18,24,25]. Th2 cytokines and proteins are regulated by genes of *IL-4* [12,13,15,16,17,18,19,20,22,26,27,28,29,30,31,32], *IL-4R* [29,32,33], *IL-5* [12,13,16,17,18,19,20,22,23,26,27], *IL-9* [17,20,22], *IL-13* family [12,13,16,17,18,19,20,22,23,25,26,27,29,31,34], *GATA3* [23,29], *STAT6* [18,23,29,31,32,34], *JAK1* [35], and *JAK3* [32,33,35] genes. Th17 and Treg cells are characterized by proteins encoded by gene of the *IL-17* family [12,19,22], *IL-21* family [22,36], and *IL-22* family [19,22]; and *FOXP3* [37,38] and *CTLA4* [39]; respectively. Table 1 shows genes of the different pathways and the number of cytosine–phosphate–guanine DNA sites (CpG) representing epigenetic marks related to these genes.

Wegmann and colleagues were the first to propose that pregnancy is a Th2-predominant condition with a down-regulation of Th1 responses [2]. The authors suggested that at the beginning of pregnancy a Th1 environment dominates in the uterus to facilitate successful implantation, then changes through midpregnancy into a Th2 condition for the longest period of the pregnancy. At the end of pregnancy, there is another up-regulation of Th1 cytokines before delivery to facilitate labor with an inflammatory environment [5,40,41]. Th17 cells are considered to promote an inflammatory response during gestation whereas Treg cells are suggested to inhibit inflammation and induce tolerance; they also play a major role in immune regulation [42,43]. One study reported that the Th17 cell counts were not different in pregnant and nonpregnant women [42,44], whereas another study showed lower Th17 cell counts in the second half of pregnancy [45]. An increase in the Th1 pathway activity (gestational age not specified) has been reported in investigations that analyzed recurrent miscarriages [46,47] or preeclampsia [48,49]. Nevertheless, some studies also reported a Th2-dominant immunity in recurrent miscarriages without specification of gestational age [50] or in early pregnancy [51], which questions the assumption that a Th2-dominant immunity exists in a normal pregnancy. Hence, the Th1/Th2 paradigm might not be sufficient to explain the changes in the immune system in a successful pregnancy, and there is a need to expand research into Th1, Th2, Th17, and Treg immune pathways. We identified potential genes in these pathways starting with the review by Saito et al. [42], including most the genes mentioned in this review; three genes were added based on more recent publications (*JAK1*, *JAK3*, *CTLA4*).

It has been suggested that T cell pathways are under epigenetic regulation, and changes in cytokine activities are linked to gene activity [52,53]. Differential methylation of CpGs represent present epigenetic marks associated with the regulation of cellular processes including gene expression and cellular differentiation [54,55]. Moreover, several reports suggest that factors increasing DNAm may help in silencing T helper differentiation [17,26,56,57].

**Figure 1:**
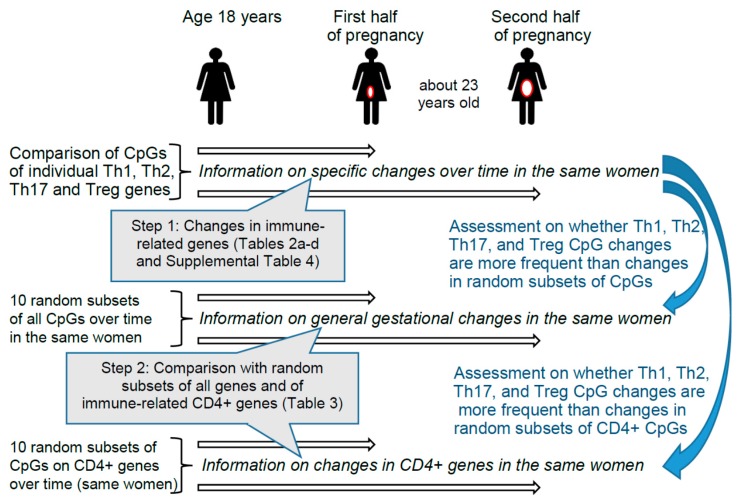
Design and steps of the epidemiologic analyses. Step 1 provides information on changes for 348 specific CpGs found in Th1, Th2, Th17, and Treg immune genes described in Table 1. In Step 1 we checked each CpG of different genes individually. In Step 2, we tested whether the 348 CpGs of genes involved in T cell pathways changed more frequently from prepregnancy to early or late pregnancy than randomly selected CpGs from non-T cell-pathways. The two comparison groups of CpGs included first, 10 subsets of randomly selected CpGs from all other non-T cell-pathways CpGs, and second, 10 subsets of randomly selected CpGs from other CD4+-genes (immune-related genes) [58].

These findings motivated us to investigate whether during gestation differential DNAm patterns can be identified in genes that encode immune markers of different T helper and Treg pathways and/or aid in their differentiation. Since immune responses keep changing throughout pregnancy, we conducted separate analyses comparing prepregnancy with the first as well as with the second half of gestation [5,40,41]. Hence, in the same women, we contrasted the CpGs of genes that are known to play a role in immune deviations during pregnancy (Table 1) with other genes that are not involved in T helper and Treg immune pathways (random subsets). Random subsets were chosen because the methylation of all CpGs may potentially be equally affected by gestational transitions. Figure 1 provides information on the two steps of the epidemiologic analyses.

## 2. Results

Of the 245 girls whose DNAm was measured at 18 years of age, the DNAm of 54 women was reassessed in the first and second halves of their pregnancy (*n* = 35 and *n* = 39 respectively), which occurred 1 to 6 years later (age at conception: 19 to 24 years). Since the four immune pathways are also involved in allergic diseases, we compared the occurrence of these diseases at the different time points. The groups of women with DNAm in the first and second halves of pregnancies did not differ with regard to asthma, eczema, atopy, and current smoking status from the women with DNAm at age 18 years (Supplemental Table S1), suggesting no selection bias. This table also shows a median age of about 23 years during pregnancy, information on body mass index, and that about 80% of the women who participated in this detailed follow-up study used folic acid supplements, or some used multivitamin drugs. Supplemental Table S2 shows the frequency of smoking during pregnancy for the two groups of participants compared to their smoking status at 18 years of age. Of the women in the first half of pregnancy (*n* = 39), 21 participants neither smoked at age 18 nor during pregnancy (84%). Five women smoked at age 18, but not during pregnancy. Among the participants in the second half of pregnancy (*n* = 35), 15 participants never smoked at age 18 nor in pregnancy (79%) and seven participants who were smokers at age 18 did not smoke during pregnancy. The estimated proportions for different cell types at age 18 and during pregnancy are provided in Supplemental Table S3. 

Table 2, Table 3, Table 4 and Table 5 show the genes and their CpGs that changed. The estimates indicate the magnitude of change in methylation from prepregnancy to pregnancy, and their *p*-values. Regarding genes in the Th1 immune pathway (Table 2 and Supplemental Table S4—descriptive information on all tested CpGs), 43 of 155 (27.7%) CpGs in the first half of pregnancy and 56 of 155 (36.1%) in the second half (Table 2) were significantly differently methylated (M-values) when compared with in the nonpregnant state. Thirty Th1 CpGs were differently methylated (Table 2) in both periods. Of the CpGs in the Th2 pathway, 27 and 26 of 77 CpGs (35.1% and 33.8%) were significantly differently methylated during the first and second halves of pregnancy, respectively (Table 3). Most CpGs in these pathways were affected similarly in the first and second half of pregnancy and all in the same direction, if identified in the first and second halves of pregnancy (higher or lower methylated, Table 2, Table 3, Table 4 and Table 5). Interestingly, five of six CpGs of the *IL13RA1* gene were changed, two with higher changes in methylation (M-value, Table 3). Of the Th17 pathway, 34 and 33 of 106 CpGs significantly changed their methylation levels (M-values) in the first and second halves of pregnancy (32.1% and 31.1%), respectively (Table 4). In 7 of 12 CpGs in the *IL21R* gene, the methylation level was altered; 6 CpGs showed a decrease in methylation; 1 CpG site was increased (M-value). The Treg pathway genes represent the smallest group; 3 and 4 of 10 CpGs were different in the first and second half of the pregnancy compared with in the nonpregnant state (Table 5). In one *FOXP3* CpG site, the methylation change was larger. The changes identified in all randomly selected CpGs (348 CpGs in 20 random subsets) are provided in Supplemental Table S4. The results adjusted for false discovery rate (FDR) are similar to the results based on the crude *p*-values presented above (Table 2, Table 3, Table 4 and Table 5).

Comparing the proportion of CpGs in the different pathways with 10 random subsets of other CpGs, in the first half of pregnancy, the Th2 immune pathway indicated the largest difference (Table 6). The risk ratios for being statistically significantly different were 1.45 times higher for the Th2 pathway compared with the average of 10 random subsets of CpGs from all genes and 1.46 times higher compared with the average of 10 random subsets of CpGs of CD4+-related genes (explained in the method section and documented in Supplemental Table S8 and S9). In other words, compared to random subsets, changes of CpGs in the Th2 pathway were 46% more likely. In second half of pregnancy, the Th1 immune pathway showed an increased risk ratio between 1.35 and 1.43 for significantly changed methylation in CpGs compared to CpGs of the two different random subsets.

Adjusting for FDR resulted in a reduction of significant CpGs in both the immune pathway genes and in genes from the two random subsets. However, the reduction of significant CpGs in random subsets was higher. Thus, the relative risk of Th2 immune pathway genes in the second half of pregnancy increases from about 1.3–1.4 (30–40% difference) to about 1.7 (70% difference, Table 6). Note that comparable effects were detected when contrasting specific T helper cell CpGs with CpGs from all genes and also with CpGs of genes related to CD4+ cells. In second half of pregnancy, when comparing CpGs in immune pathway genes with the reference CpGs, the Th1 immune pathway showed an increased risk ratio between 1.68 and 1.72 for significantly changed methylation in CpGs contrasted with CpGs of the two different random subsets (all CpGs and CpGs related to CD4+ cells). Adjusting for multiple testing also resulted in higher relative risks of significantly changed CpGs in the Th2 pathway (Table 6). CpGs in the Th2 pathway were 1.74 to 1.77 times more likely to have changed from the prepregnancy to the pregnancy status. However, compared to the reference subsets, the 95% confidence interval of statistically significantly changed CpGs in the Th17 pathway included the value one and thus was not statistically different. 

The position of the significantly different CpGs relative to gene structure shows some interesting differences (Table 7). As expected, since the selection was based on genes, there were no CpG sites in intergenic regions of the CD4+-related genes as well as the ones from Th1/Th2/Th17/Treg pathways. However, the reference group of CpGs from the whole genome had 21.9% CpGs from intergenic regions. As a consequence of the higher number of intergenic CpGs, the proportion of CpGs located in the promoter region is lower in the randomly selected CpGs from the whole genome than in the reference group of CpGs from genes expressed on CD4+ cells and in CpGs from the Th1/Th2/Th17/Treg pathway genes. For this reason, we compared the proportion of significant CpGs in the reference groups with and without CpGs in the intergenic regions, as shown in Supplemental Table S5. We found comparable proportions of significantly changed CpGs, indicating that there is no bias based on the inclusion of intergenic CpGs in the whole genome reference group. Supplemental Table S6 shows whether significant CpG methylation changes of the same direction (increase or decrease) clustered within 1500 base pairs up- or downstream of one significantly altered CpG. There is no clear pattern regarding the spatial clustering of significant CpG sites.

We also compared the number and proportion of significant CpGs in the group of 20 participants who participated three times (before pregnancy, first half and second half of gestation) with 39 participants in the first half of pregnancy and with 35 participants in the second half of pregnancy. The nonpregnant state at age 18 was used as the reference in both scenarios. The proportion of significantly changed CpG sites among the two groups were not statistically different from each other, neither in the first nor the second half of pregnancy, as shown in Supplemental Table S7. However, the proportion of significantly changed CpG sites was slightly lower in the group of women who participated three times. 

## 3. Discussion

To our knowledge, this is the first study comparing changes in DNAm of CpGs in Th1, Th2, Th17, and Treg pathway genes from before pregnancy to during the first and second halves of pregnancy. In the first half of pregnancy, changes in methylation levels were more frequent among Th2 pathway genes, while in the second half, changes in methylation of CpGs of the Th1 pathway were more frequent compared to those of randomly selected CpGs. Also, in the second half of pregnancy, more changes in CpGs in Th2 pathway genes are seen after adjustment for multiple testing. The findings were similar and independent on whether we compared the immune pathway CpGs with random subsets of all CpGs or with random subsets of CpGs of genes found on CD4+ cells (immune-cell-related). We did not consider the direction of the methylation changes, just its presence, since, depending on the methylation of specific regions, lower and higher methylation of different regions may have the same functional effects (e.g., gene expression).

One limitation of our study is that the CpGs were taken from white blood cells, but not from specific cell types, since we could not separate the cells and costs for DNAm analyses would have been multiplied by the number of different cells. To overcome this limitation, we deconvoluted the analyses of the CpGs by adjusting for cell types. In addition, we also took 15 surrogate variables into account—a procedure which has been demonstrated to address latent subjects which may affect DNAm [59]. We did not attempt to replicate the DNAm measurements based on the Illumina 450 K array with other methods such as pyrosequencing since this array has been shown to be technically reliable in multiple studies [60,61,62,63,64,65,66,67]. Another limitation of this study is that blood subsets could not be collected in all three trimesters; however, the separation of gestation into trimesters is arbitrary. We therefore decided to analyze changes in methylation from age 18 years (prepregnancy) to the first and second halves of pregnancy. The subsets of pregnant women are part of a whole birth cohort; however, it only represents a Caucasian population. Hence, results might differ for populations of other races and ethnicities. Genes of Th1/Th2/Th17 and Treg pathways were selected based on prior study reports, excluding genes with contradictive findings that could not be allocated to one pathway (Table 1). 

One strength lies in comparing the same women before and during pregnancy using repeated measurements of DNAm (age 18 and during pregnancy). A second strength is the design that does not compare women who conceived with women who did not conceive, which would require taking a wide range of confounding into account. Since we focus on intra-individual changes, we also did not need to adjust for genetic polymorphisms, which in turn influence the potential of the methylation of specific CpG sites. Against that, we contrasted CpGs of genes that are known to play a role in immune deviations during pregnancy with random subsets of genes that are not involved in T helper and Treg immune pathways (random subsets). We used random subsets because the methylation of all CpGs may potentially be equally affected by gestational transitions, providing a contrast with immune pathway genes. The advantage of this design is that potential confounding factors such as aging are implicitly taken into account when comparing different sets of CpGs in the same mother. A limitation of our study is the small sample size or a lower statistical power; however, we found a large number of methylation changes in the four immune pathway genes. Nevertheless, we may not have identified weaker changes in the four immune pathway genes as statistically significant. 

Gestational immunity has been suggested to be a Th2-predominant condition with a down-regulation of Th1 [2]. The results presented here shows changes in the DNAm of Th2 pathway genes in the first and second halves of pregnancy and changes in the DNAm of Th1 pathway genes during the second half of pregnancy. In particular, CpGs on the *IL13RA1* (Th2 pathway) showed a number of changes, which were large compared with those of other CpGs. Frequent changes in DNAm of Th2 pathway genes in the first and second halves of pregnancy and frequent changes in DNAm of Th1 pathway genes during the second half of pregnancy suggest that both Th1 and Th2 pathways are involved in the gestational immune bias. Moreover, experimental studies indicated that DNAm of genes related with T helper cell differentiation may induce polarization towards or away from an allergic phenotype. Thus, focusing on changes in methylation in these pathways during gestation may guide the future detection of predictive markers for allergy. 

The Th17 pathway is considered to establish a protective immune response in pregnancy [42]; however, its role has not yet been clearly established. One study reported a lower count of Th17 cells in the second half of pregnancy [45], whereas another study showed a similar frequency for Th17 cells during pregnancy compared to that in nonpregnant women. Although we do not have cell counts, in the second half of pregnancy our results suggest some changes in methylation of CpGs in Th17 pathway genes that may need future investigation. In particular, CpGs on the *IL21R* gene may need further consideration (Table 4). We also tested for the direction of change and position of the significant CpGs (Supplemental Table S6), but could not detect a clear overall pattern independent of the genes. The relation between the direction of methylation changes and the position of the CpGs on the genes and their effect on gene expression needs further investigation.

In conclusion, in Th1 pathway CpGs, comparing the prepregnancy with the pregnancy state, we found 1.68 to 1.72 times higher relative risk of change in the second half of pregnancy. CpGs in the Th2 pathway were 1.74 to 1.77 times more likely to have changed from the prepregnancy to the late pregnancy state. We did not identify any other study comparing prepregnancy with pregnancy status of DNAm. Hence, replications are warranted. Future studies should also examine whether the reported changes in methylation of maternal blood can also be found in cord blood and are related to allergic manifestations in offspring. Better knowledge of the mechanisms of Th1 and Th2 pathway regulation in pregnancy may contribute to understanding of intra-uterine origin of allergic diseases and will provide guidance to initiate preventive screening strategies for early detection of allergic diseases in offspring. 

## 4. Participants and Methods

### 4.1. The Isle of Wight Birth Cohort

The Isle of Wight Whole Population Birth Cohort was established in 1989 in United Kingdom, to prospectively study the natural history of asthma and allergic conditions. All newborns were recruited into the study in 1989 and have being followed to date. For analyses of DNAm, blood samples were collected at age 18 years and again during the first (8–21 weeks) and the second (22–38 weeks) halves of pregnancy. Questionnaires were completed at age 18 and during pregnancy and gathered information including smoking, diet, and medication, and asthma, eczema, and atopy status. The 1989 birth cohort has been described elsewhere [57,68]. Female participants of the birth cohort have been followed through their pregnancies occurring between years 2011–2015.

### 4.2. Ethics

Ethics approval was given by the Isle of Wight Local Research Ethics Committee before recruiting participants between January 1989 and February 1990. Permission was granted for all follow-ups as well as collection of samples for genetic studies. Written informed consent was obtained from all participants before they participated in the study. The investigation was approved by the Isle of Wight, Portsmouth and SE Hampshire Local Research Ethics Committee (Research Ethics Committee reference number: 09/H0504/129; 4 December 2009). At the University of Memphis, the internal review board approved the project (FWA00006815, 7 December 2012). 

### 4.3. Study Design

We measured DNAm in white blood cells in women at 18 years of age (nonpregnant state) and in the same women in the first and second halves of pregnancy. We hypothesized that, compared to a preceding nonpregnant state, the methylation of genes that code for immune markers of Th1, Th2, Th17, and Treg are more likely to change during pregnancy than any randomly selected set of nonpathway CpG sites. The analyses comprise two main and three supplementary steps. First, we compared the DNAm of all CpG sites of the selected 48 Th1, Th2, Th17, and Treg pathway genes measured separately during the first and second halves of pregnancy with DNAm measured at 18 years of age (nonpregnant) using repeated measurement models. Second, since DNAm of other genes may also change with pregnancy, we examined whether statistically significant changes in methylation levels of Th1, Th2, Th17, and Treg pathway CpGs were more frequent than changes in ten randomly selected subsets of CpGs (Supplemental Table S8). Since contrasts with one random subset may depend on the particular subset selected, we repeated the assessment in 10 random subsets. Third, as Th1, Th2, Th17, and Treg pathway genes are expressed in blood cells, but a random subset of all genes may not represent blood cells, we added a second comparison group with 10 random subsets of genes identified in CD4 (cluster of differentiation 4) blood-borne immune cells [58] (Supplemental Table S9). Fourth, since Th1, Th2, Th17, and Treg pathway genes as well as the CD4+ random subset do not include CpGs from intergenic regions, to ensure that inclusion of the intergenic regions did not bias our findings, we compared the two reference groups with CpGs excluding intergenic regions. Fifth, to make sure that the observed changes in DNAm are not explained by changes in blood cell counts between age 18 and the first and second halves of pregnancy, we adjusted for the respective cell mixtures. 

We nested a follow-up study of women in the Cohort who became pregnant to examine and compare changes in DNAm of CpGs in Th1, Th2, Th17, and Treg pathway genes from the nonpregnant state at age 18 years to the first and second halves of pregnancy. Genes encoding components of the Th1, Th2, Th17, and Treg pathways were selected based on published journal articles (Table 1) and their CpGs were selected from the Illumina 450 K array manifest file http://support.illumina.com/downloads/humanmethylation450_15017482_v1-2_product_files.html (last accessed 5 February 2018).

The main analysis was divided into two parts. First, we performed repeated measurement analyses, comparing the methylation of specific Th1, Th2, Th17, and Treg pathway genes at 18 years of age with the methylation levels of the same CpGs in the same women during the first half of pregnancy for a group of 39 women. General linear models for repeated measurements were used to determine statistically significant changes in methylation of CpGs. The same procedure was applied for the 35 women who had their DNAm assessed at age 18 and during the second half of pregnancy. The number of CpGs with statistically significant changes (*p* ≤ 0.05) was determined. Twenty participants of the sub-subsets of 39 and 35 pregnant women, also included in the 245 nonpregnant participants (at age 18 years), had DNAm measurements at all three time points. Thus, to determine whether results are altered when we focused on these 20 common participants, we additionally repeated the analyses of the time effect on DNAm (prepregnancy to pregnancy) for the subset of 20 women.

Since the methylation of other genes may also change with pregnancy, in the second part of the analyses, we used general linear models to assess methylation changes in randomly selected CpGs from the whole genome between the two time periods (age 18 years and first or second halves of pregnancy, respectively). To reduce the probability that any detected difference in DNAm between the T cell pathways and the randomly selected reference subsets is due to chance, we repeated the random sampling 10 times. That is, 10 random subsets of 348 CpGs each (which is equal to the number of total CpGs in the four pathways) were selected and used as a reference set from the complete list of CpGs. Another 10 random subsets of 348 CpGs each were selected from a list of CD4+ genes [58] and used as a second reference set. The latter reference more likely reflects genes active in blood cells. The results of the analysis of random subsets informed whether DNAm changes are more likely to occur in CpGs of T cell pathways (Th1, Th2, Th17, and Treg) during pregnancy. To this end, we compared the risks of changes from prepregnancy to pregnancy in T cell pathway CpGs with the risk of changes in the random subsets of CpGs using log–linear models and estimated risk ratios.

### 4.4. DNA Methylation

A standard salting out procedure [69] was used to extract DNA from blood samples. One microgram of DNA from each sample was bisulfite converted using the EZ-96 DNAm kit (Zymo Research, Irvine, CA, USA), and genome-wide DNAm was measured using the Illumina Infinium HumanMethylation450 beadchip (Illumina, Inc., San Diego, CA, USA). Methylation data were extracted from image data files using the Methylation module of Genome Studio software and were preprocessed using the IMA package [70] executed in the R statistical computing package. The methylation level was measured by β-values which represent the proportion of methylated (M) over the sum of methylated and unmethylated (U) allele intensities (β = M/[c + M + U]), where c is a constant to prevent dividing by zero [71]. Logit-transformed β-values (M-values) were used for these differential methylation analyses as β-values have severe heteroscedasticity [72].

The raw data of DNAm were preprocessed to achieve high quality for data analyses. The Bioconductor IMA (Illumina methylation analyzer) package31 and the ComBat32 package were used to remove background noise, adjust for interarray variation, perform peak correction, and to remove batch effects. In addition, CpGs with probe SNPs (single nucleotide polymorphisms) were removed from the list of all CpG sites if their Minor Allele Frequency (MAF) was larger than 1% (*N* = 89,678; http://support.illumina.com/downloads/humanmethylation450_15017482_v1-2_product_files.html, last accessed 5 February 2018). Probe SNPs are single-nucleotide polymorphisms within the probe, which may interfere with DNAm measurement. Due to these procedures, the number of eligible CpG sites was reduced to 274,710 probes (approximately 60% of the original number).

We then tested methylation changes in 348 CpGs annotated to genes in the Th1, Th2, Th17, and Treg pathways (Table 1). For Th1, we examined CpGs of the following genes*: IFN-γ* family, *TNF-α* family, *IL-2*, *IL-12* family, and *IL-12RB1*, *IL-12RB2*; for the Th2 pathway, CpGs of the *IL-4*, *IL-4R*, *IL-5*, *IL-9*, *IL-13* family, *GATA3*, *STAT6*, *JAK1*, *JAK3* and *IL1RL1*; for Th17, *IL-17* family, *IL-21* family, and *IL-22* family; and for the Treg pathway, *FOXP3* and *CTLA4* genes. The final numbers of CpGs were 155 CpGs (19 genes) for Th1, 77 CpGs (12 genes) in Th2, 106 CpGs (15 genes) in Th17, 10 CpGs (2 genes) for Treg; and an identical number of 348 CpGs in each randomly selected reference set.

### 4.5. Variables Used for the Description of the Population Samples

Since some of the genes in the four immune pathways are also involved in allergic diseases, we briefly described our cohort with regard to these diseases. At 18 years of age, information about asthma was collected using the International Study of Asthma and Allergies in Childhood (ISAAC) questionnaire [73]. Asthma was defined as history of physician-diagnosed asthma, combined with wheezing or whistling in the chest in the last 12 months and/or asthma treatment in last 12 months. Eczema was defined as chronic or chronically relapsing itchy dermatitis lasting more than six weeks with characteristic morphology and distribution [74], following Hanifin and Rajka criteria [75]. At 18 years, to define atopy or allergic sensitization, regardless of symptoms, skin prick tests (SPT) were performed with a standard battery of 13 common allergens (ALK-Albello, Horsholm, Denmark). Inhalant allergens tested were house dust mite, cat, dog, *Alternaria alternata*, *Cladosporium herbarium*, grass pollen mix, and tree pollen mix; food allergens tested were cows’ milk, soya, hens’ egg, peanut, and cod. Atopy or allergic sensitization was defined as having a response to at least one allergen of mean wheal diameter 3 mm greater than the negative control. Participants were asked about their current smoking status at age 18 years, twice during pregnancy, and again after delivery. These responses were grouped into the following categories, “never”, “early in pregnancy”, “transient” (occasional), and “throughout pregnancy”.

### 4.6. Statistical Analyses

The baseline to assess changes in DNAm was 245 women of the birth cohort, who had DNAm at age 18 years. To check whether the two study samples (39 women with DNAm measured in the first half of pregnancy, and 35 women with DNAm measured in the second half of pregnancy (total of 54 women)) are similar to characteristics of all women who had their DNAm measured at 18 years of age (*n* = 245), the characteristics of those 54 women were compared against the 245 women (total number of women with DNAm data at age 18 years) in the cohort using the Chi-square test.

The Bioconductor IMA (Illumina methylation analyzer) package and the ComBat package [76] were used to remove background noise, adjust for interarray variation, perform peak correction, quantile normalization, and remove batch effects (7 batches) [70,77]. DNAm levels for each CpG were estimated as the proportion of intensity of methylated (M) over the sum of methylated (M) and unmethylated (U) probes, β = M/(c + M + U), with c being a constant to prevent dividing by zero. We focused on the 22 autosome excluding all CpGs on the sex chromosomes. Methylation determined in whole blood can distort the comparison of DNAm between the prepregnancy and the pregnancy states since it contains different cell types with different methylation levels. To overcome this limitation, we conducted two deconvolution steps to adjust for cell mixture effects on the methylation of all CpGs (a non-reference-based surrogate variable analysis (SVA) followed by cell type adjustment). The non-reference-based method utilizes singular value decomposition as conducted in surrogate variable analysis (SVA) [59]. The fifteen most important surrogate variables, identified in SVA, were then used in regression models, separately for nonpregnant data and for the data of the first and second halves of pregnancy, to estimate the part of the methylation of the CpGs (the residuals) that was not influenced by the 15 surrogate variables. To additionally adjust for cell types (B cells, CD4+ T cells, CD8+ T cells, eosinophils, granulocytes, monocytes, and natural killer cells), we estimated the cell type proportions for each sample using Bioconductor [78], the R package “minfi” [79], which is based on reference values of cell-type-specific CpGs [80]. Both SVA and cell type adjustment were used since the latter, based on data of men, may not provide sufficient adjustment for women. The SVA adjustments were performed in R-3.1.0. 

General linear models with repeated measurements (measured each time at age 18 and during pregnancy) were then applied using the residual DNAm to test whether there were statistically significant changes between the prepregnancy and pregnancy states while adjusting cell type proportions estimated each time. Time and cell type proportions were used as fixed effects. Unstructured covariance was used to allow for heterogeneous variances at each time and heterogeneous covariance between two time points. General linear models and log-linear models were done in SAS 9.3. 

The cell type proportions then were additionally used in repeated measurement models of the residual DNAm to test whether there were statistically significant changes between the prepregnancy and pregnancy states. Hence, we deconvoluted the methylation of CpGs twice using residuals after adjusting for surrogate variables (SV) and estimated cell type proportions in the repeated measurement model. 

To assess changes in DNAm between age 18 years and first and second halves of pregnancy in the four immune pathways and in the reference CpGs outside these pathways, we compared the methylation of Th1, Th2, Th17, and Treg CpGs and randomly selected CpGs at these two time points. General linear models, adjusted for cell type proportions, were applied to the SV-adjusted residuals at each time point as the response variable with time as the independent variable. Multiple testing was adjusted by controlling false discovery rate (FDR) [81].

Once we identified, in general linear models, CpGs whose methylation was significantly different between prepregnancy and pregnancy, in a second step, we examined whether DNAm changes related to pregnancy in the four immune pathways are more likely to occur in the identified CpGs compared to in CpGs in references. Two reference sets were employed: the 10 randomly selected subsets of CpGs from the complete list of all CpGs (3480 of the total of 274,710 CpGs) and another 10 random subsets of 3480 CpGs from a set 9620 CpGs of CD4+ genes [58] (each having 348 CpGs). The latter set allowed a comparison of immune pathways in blood-borne cells with genes of blood-borne CD4+ cells. Log-linear models were then applied to test whether significant changes in DNAm as the response variable were more frequent in the four immune pathways compared to in the random reference set. To ensure that the results do not depend on a single random selection, we repeated the procedure 10 times with multiple sets of reference CpG sites, randomly selected from all 274,354 CpG or from 9620 CD4+-related CpGs with each set composed of the same number of CpGs as the sum of CpGs from all the four pathways (348 CpGs). This analysis provides sets of the T helper pathway CpGs and of CpGs from random subsets that were significantly affected by pregnancy compared to the prepregnancy status. We then estimated the risks on whether Th2 pathway CpGs were more often statistically significantly changed than random subsets of CpGs using the GENMOD procedure (SAS 9.3). The estimated risk ratios show how the number of CpGs that are significantly different in the respective pathway (Th1, Th2, Th17, or Treg) is larger compared with the number of significantly different CpGs identified in random subsets. These risk ratios and their 95% confidence limits for the 10 random subsets were, at that point, combined using the median proportions of significant changes, separate for the two different sets (random CpGs from the whole genome and random CpGs from CD4+ T-cell-specific genes). The estimation of risk ratios was repeated for the CpGs that remained statistically significant after adjustment for false discovery rate (FDR) in the four pathways and in the random subsets. 

Finally, we inspected the direction of change for CpGs in the promoter, the 5′UTR, body region, and the 3′UTR region of the various immune pathway genes. Finally, to determine whether statistically significant methylation changes of CpGs were clustered, we tested for each CpG site with a significant change, whether adjacent CpGs, 1500 base pairs upstream, and 1500 base pairs downstream also changed significantly from the nonpregnant to the pregnant state. The significance level for all models was set at *p* ≤ 0.05.

## Figures and Tables

**Table 1:** Genes considered for T cell pathways in the study and their numbers of cytosine–phosphate–guanine (CpG) sites.

Pathway	Number of Genes	Genes Coding Cytokines, Proteins, or Receptors	Number of CpG Sites
Th1	19	*IFN-γ* family [12,13,14,15,16,17,18,19,20,21,22,23] *TNF-α* family [12,13,14,15,16,17,18,19,20,21,22,23] *IL-2* [12,13,14,17,20,22,23] *IL-12* family [13,18,24]	155
Th2	12	*IL-4* [12,13,15,16,17,18,19,20,22,26,27,28,29,30,31,32] *IL4R* [29,32,33] *IL-5* *5* [12,13,16,17,18,19,20,22,23,26,27] *IL-9* [17,20,22] *IL-13* family [12,13,16,17,18,19,20,22,23,25,26,27,29,31,34] *GATA3* [23,29] *STAT6* [18,23,29,31,32,34] *JAK1* [35] *JAK3* [32,33,35] *IL1RL1* [12,19,22]	77
Th17	15	*IL17* family [12,19,22] *IL21* family [22,36] *IL22* family [19,22]	106
Treg	2	*FOXP3* [37,38] *CTLA4* [39]	10

**Table 2:** Th1 CpGs showing statistically significant changes (*p* ≤ 0.05) during first and second halves of pregnancy ^a^.

Th1 Gene Names	Total Number of CpGs	Significant CpGs in First Half of Pregnancy ^b^					Significant CpGs in Second Half of Pregnancy ^c^				
		No. of Significant CpGs	CpG Sites	Parameter Estimates	*p*-Value	FDR-Adjusted *p*-Value	No. of Significant CpGs	CpG Sites	Parameter Estimates	*p*-Value	FDR-Adjusted *p*-Value
*IFNG*	5	1	cg01940810	0.15	0.034	0.145	1				
								cg26227465	0.13	0.014	0.056
*IFNGR1*	3	1	cg26668632	−0.14	0.045	0.163	1				
								**cg07401792**	−0.25	0.011	0.044
*IFNGR2*	10	3	**cg08173915**	−0.49	1.00 × 10^−32^	<1.0 × 10^−6^	4	**cg08173915**	−0.44	<1 × 10^−16^	<1.0 × 10^−6^
			**cg17356733**	−0.5	4.44 × 10^−16^	<1.0 × 10^−6^		**cg17356733**	−0.43	3.48 × 10^−11^	<1.0 × 10^−6^
			**cg22669060**	−0.49	1.01 × 10^−12^	<1.0 × 10^−6^		**cg22669060**	−0.47	1.05 × 10^−10^	<1.0 × 10^−6^
								cg27469991	−0.15	0.018	0.066
*IL12A*	7	3	**cg09362366**	−0.56	6.60 × 10^−4^	0.004	2				
			**cg20515136**	−0.31	4.34 × 10^−6^	1.1 × 10^−5^		**cg20515136**	−0.5	1.16 × 10^−7^	<1.0 × 10^−6^
			**cg25829945**	−0.24	0.041	0.150		cg25829945	−0.18	0.039	0.113
*IL12B*	9	1	**cg06111286**	−0.39	2.31 × 10^−5^	2.4 × 10^−5^	1	**cg06111286**	−0.45	3.16 × 10^−9^	<1.0 × 10^−6^
*IL12RB1*	6	1	**cg12123019**	−0.5	1.35 × 10^−4^	0.001	2	**cg12123019**	−0.33	6.963 × 10^−4^	0.005
								cg18307303	−0.14	0.016	0.059
*IL12RB2*	10	7	**cg02566391**	0.46	1.48 × 10^−12^	<1.0 × 10^−6^	8	**cg02566391**	0.49	<1 × 10^−16^	<1.0 × 10^−6^
			cg06952660	−0.29	0.030	0.140		**cg06952660**	−0.32	3.54 × 10^−4^	0.003
			**cg09018107**	0.48	7.67 × 10^−10^	<1.0 × 10^−6^		**cg09018107**	0.37	2.44 × 10^−6^	2.0 × 10^−6^
			**cg12633410**	0.21	0.031	0.140		cg12633410	0.16	0.025	0.081
			**cg14849855**	0.32	1.53 × 10^−7^	<1.0 × 10^−6^		**cg14849855**	0.19	0.004	0.021
			**cg19745415**	−0.49	4.40 × 10^−4^	0.003		**cg19745415**	−0.41	0.003	0.016
			**cg20253742**	−0.53	7.27 × 10^−5^	0.001		**cg20253742**	−0.25	0.0023	0.012
								cg11132246	−0.27	0.021	0.073
*IL2*	1	0					0				
*IL2RA*	2	0					0				
*IL2RB*	4	1	**cg21307484**	0.27	6.63 × 10^−5^	0.001	3	**cg21307484**	0.18	0.002	0.011
								**cg24509815**	0.18	2.13 × 10^−9^	<1.0 × 10^−6^
								**cg26757673**	0.23	4.09 × 10^−7^	<1.0 × 10^−6^
*TNF*	11	4	**cg01360627**	0.16	0.006	0.031	6	**cg01360627**	0.3	2.42 × 10^−11^	<1.0 × 10^−6^
			**cg04425624**	−0.27	0.002	0.011					
			**cg10650821**	−0.33	1.43 × 10^−4^	0.001		**cg10650821**	−0.3	7.45 × 10^−4^	0.005
			**cg10717214**	−0.25	0.002	0.011					
								cg17755321	0.15	0.027	0.088
								cg15989608	0.21	0.043	0.122
								**cg23384708**	0.22	6.21 × 10^−5^	0.001
								cg26736341	0.14	0.032	0.099
*TNFAIP1*	5	1	**cg22640868**	−0.5	2.29 × 10^−12^	<1.0 × 10^−6^	4	**cg22640868**	−0.46	1.27 × 10^−13^	<1.0 × 10^−6^
								cg26663469	0.16	0.033	0.101
								**cg11814826**	−0.24	0.010	0.031
								cg13290523	−0.18	0.031	0.097
*TNFAIP2*	8	2	cg03021690	0.12	0.036	0.145	4	**cg03021690**	0.14	0.002	0.015
			cg13144594	0.23	0.012	0.058		cg13144594	0.26	0.016	0.059
								cg03572388	−0.12	0.049	0.135
								**cg04264002**	0.14	0.007	0.033
*TNFAIP3*	13	4	**cg06779945**	0.17	0.039	0.049	2				
			**cg08919597**	−0.84	0.0000	<1.0 × 10^−6^		**cg08919597**	−0.43	0.003	0.018
			cg12200164	−0.19	0.035	0.145					
			cg18264753	−0.13	0.018	0.085					
								**cg12214665**	−0.14	0.011	0.044
*TNFAIP6*	4	1	**cg03406844**	−0.49	1.79 × 10^−12^	<1.0 × 10^−6^	1	**cg03406844**	−0.51	4.44 × 10^−16^	<1.0 × 10^−6^
*TNFAIP8*	22	9	**cg00524900**	−0.71	1.03 × 10^−9^	<1.0 × 10^−6^	7	**cg00524900**	−0.88	<1 × 10^−16^	<1.0 × 10^−6^
			**cg01057573**	−0.73	<1 × 10^−16^	<1.0 × 10^−6^		**cg01057573**	−0.72	<1 × 10^−16^	<1.0 × 10^−6^
			**cg03665078**	−0.21	0.001	0.007		**cg03665078**	−0.17	0.009	0.040
			**cg07398791**	−0.31	4.40 × 10^−6^	1.1 × 10^−5^		**cg07398791**	−0.35	1.83 × 10^−7^	<1.0 × 10^−6^
			**cg11846226**	0.22	1.34 × 10^−4^	0.001					
			cg12148675	−0.18	0.037	0.145					
			cg15408889	−0.2	0.010	0.053		**cg15408889**	−0.2	0.010	0.042
			**cg21239001**	−0.45	6.10 × 10^−12^	<1.0 × 10^−6^		**cg21239001**	−0.49	8.88 × 10^−16^	<1.0 × 10^−6^
			**cg07086380**	0.22	2.67 × 10^−4^	0.002		**cg07086380**	0.27	6.82 × 10^−5^	0.001
*TNFAIP8L*	30	4	**cg21544402**	−0.33	4.61 × 10^−4^	0.003	10				
			**cg05503460**	−0.26	2.16 × 10^−4^	0.002		**cg05503460**	−0.3	3.251 × 10^−5^	3.1 × 10^−5^
			cg12122631	−0.15	0.039	0.149					
			cg23343680	−0.15	0.034	0.145		**cg23343680**	−0.15	0.006	0.001
								**cg02436098**	−0.2	0.007	0.032
								cg11708963	−0.17	0.0237	0.079
								cg22754389	−0.15	0.036	0.108
								**cg23612220**	−0.44	0.005	0.026
								**cg02233197**	0.18	0.008	0.038
								**cg02346713**	−0.25	1.37 × 10^−5^	1.2 × 10^−5^
								cg03454639	0.22	0.024	0.081
								cg22038124	0.1	0.039	0.113
Total	155	43					56				

^a^ CpGs with statistically significant differences after applying false discovery rate (FDR) tests are bolded; ^b^ Changes in methylation from age 18 years to first half of pregnancy (8–21 weeks) for Treg pathway gene CpGs; **^c^** Changes in methylation from age 18 years to second half of pregnancy (22–38 weeks) for Treg pathway gene CpGs.

**Table 3:** Th2 CpGs showing statistically significant changes (*p ≤* 0.05) during first and second halves of pregnancy ^a^.

Th2 Genes	Total Number of CpGs	Significant CpGs in First Half of Pregnancy ^b^					Significant CpGs in Second Half of Pregnancy ^c^				
		No. of Sign. CpGs	CpG Sites	Parameter Estimates	*p*-Value	FDR-Adjusted *p*-Value	No. of Sign. CpGs	CpG Sites	Parameter Estimates	*p*-Value	FDR-Adjusted *p*-Value
*GATA3*	17	6	cg00463367	0.18	0.025	0.095	4	**cg00463367**	0.29	2.94 × 10^−6^	2.0 × 10^−6^
			cg01255894	−0.27	0.014	0.055					
			**cg03669298**	−0.32	1 × 10^−4^	0.001		**cg03669298**	−0.28	2 × 10^−4^	0.001
			**cg10008757**	−0.15	0.007	0.034		**cg10008757**	−0.2	0.006	0.027
			**cg11430077**	0.38	0.001	0.005					
			**cg22770911**	0.24	2 × 10^−4^	0.002		cg22770911	0.12	0.021	0.074
*IL13*	4	1	**cg15329179**	0.19	0.005	0.024	1				
								cg13566430	−0.13	0.042	0.130
*IL13RA1*	6	5	**cg01080862**	1.8	1.27 × 10^−9^	<1.0 × 10^−6^	5	**cg01080862**	1.77	1.41 × 10^−12^	<1.0 × 10^−6^
			**cg22817042**	1.19	6.81 × 10^−10^	<1.0 × 10^−6^		**cg22817042**	0.94	7.48 × 10^−10^	<1.0 × 10^−6^
			cg23508470	−0.13	0.039	0.126		**cg23508470**	−0.16	0.012	0.047
			**cg25968748**	1.06	9.49 × 10^−13^	<1.0 × 10^−6^		**cg25968748**	0.76	3.99 × 10^−8^	<1.0 × 10^−6^
			**cg27501007**	1.92	<1 × 10^−16^	<1.0 × 10^−6^		**cg27501007**	1.76	<1 × 10^−16^	<1.0 × 10^−6^
*IL13RA2*	2	1	**cg03244736**	−0.93	0.001	0.005	1	**cg03244736**	−0.53	0.013	0.049
*IL1RL1*	4	2	**cg11916609**	−0.35	0.002	0.014	1	**cg11916609**	−0.36	6.14 × 10^−7^	<1.0 × 10^−6^
			cg17738684	0.15	0.048	0.138					
*IL4*	1	0					0				
*IL4R*	8	4	**cg01165142**	−0.34	1.63 × 10^−6^	2.0 × 10^−6^	4	**cg01165142**	−0.39	9.78 × 10^−8^	<1.0 × 10^−6^
			cg05903710	−0.14	0.0427	0.126					
			**cg16649560**	−0.54	5.50 × 10^−13^	<1.0 × 10^−6^		**cg16649560**	−0.53	<1 × 10^−16^	<1.0 × 10^−6^
			cg26937798	−0.32	0.042	0.126		cg26937798	−0.3	0.033	0.112
								**cg05729093**	0.21	0.008	0.035
*IL5*	2	0					1	**cg16184131**	−0.39	4.52 × 10^−8^	<1.0 × 10^−6^
*IL5RA*	7	3	cg01310029	0.19	0.034	0.120	3				
			**cg23032421**	0.56	5.21 × 10^−4^	0.004		**cg23032421**	0.58	7.72 × 10^−12^	<1.0 × 10^−6^
			**cg23828301**	−0.68	2.64 × 10^−13^	<1.0 × 10^−6^		**cg23828301**	−0.67	<1 × 10^−16^	<1.0 × 10^−6^
								**cg08404225**	0.25	0.001	0.005
*IL9*	3	0					0				
*JAK1*	11	3	**cg00153395**	−0.22	5.77 × 10^−6^	1.0 × 10^−5^	3	**cg00153395**	−0.38	6.29 × 10^−10^	<1.0 × 10^−6^
			cg12444684	−0.16	0.029	0.107					
			cg26315985	−0.27	0.013	0.055					
								cg07798602	0.14	0.014	0.052
								**cg25020373**	−0.28	0.001	0.003
*JAK3*	7	1	cg06655414	0.34	0.039	0.126	2				
								cg02285920	−0.27	0.049	0.145
								**cg25623545**	−0.25	0.008	0.035
*STAT6*	5	1	cg12693595	0.16	0.012	0.053	1				
								cg25157914	−0.15	0.036	0.114
Total	77	27					26				

^a^ CpGs with statistically significant differences after applying false discovery tests are bolded. ^b^ Changes in methylation from age 18 years to first half of pregnancy (8–21 weeks) for Th2 pathway gene CpGs. **^c^** Changes in methylation from age 18 years to second half of pregnancy (22–38 weeks) for Th2 pathway gene CpGs.

**Table 4:** Th17 CpGs showing statistically significant changes (*p ≤* 0.05) during first and second halves of pregnancy ^a^.

Th17 Gene Names	Total Number of CpGs	Significant CpGs in First Half of Pregnancy ^b^					Significant CpGs in Second Half of Pregnancy ^c^				
		No. of Sign. CpGs	CpG Sites	Parameter Estimates	*p*-Value	FDR-Adjusted *p*-Value	No. of Sign. CpGs	CpG Sites	Parameter Estimates	*p*-Value	FDR-Adjusted *p*-Value
*IL17A*	2	1	cg05884768	−0.21	0.046	0.155	0				
*IL17B*	7	1	**cg01579636**	0.21	0.002	0.014	2	**cg01579636**	0.13	0.011	0.047
								cg05860978	−0.12	0.014	0.059
*IL17C*	5	3	cg08155347	−0.21	0.012	0.058	2				
			**cg26686608**	0.14	3.27 × 10^−4^	0.003		**cg26686608**	0.14	0.009	0.042
			**cg27132152**	0.18	0.009	0.050		**cg27132152**	0.23	0.004	0.020
*IL17D*	13	1	cg09985351	0.2	0.047	0.155	2				
								**cg12475590**	−0.27	0.002	0.017
								**cg02792322**	−0.17	0.006	0.031
*IL17F*	5	0					0				
*IL17RA*	8	5	cg01085328	0.17	0.011	0.055	4				
			**cg01760983**	−0.65	1.23 × 10^−4^	0.001		cg01760983	−0.24	0.039	0.130
			**cg02866761**	−0.16	0.001	0.009					
			**cg16389078**	−0.52	2.72 × 10^−8^	<1.0 × 10^−6^		**cg16389078**	−0.36	0.001	0.005
			cg19901866	0.24	0.032	0.118					
								cg13595439	0.15	0.015	0.061
								**cg15502903**	−0.19	1.76 × 10^−4^	0.002
*IL17RC*	2	0					0				
*IL17RD*	15	4	cg00770158	−0.09	0.029	0.110	5				
			**cg01797381**	−0.29	0.005	0.027		**cg01797381**	−0.32	4.18 × 10^−4^	0.004
			**cg03435901**	−0.48	5.82 × 10^−5^	0.001		**cg03435901**	−0.4	0.003	0.020
			**cg10882522**	−0.28	0.001	0.009		cg10882522	−0.18	0.019	0.073
								**cg09429700**	0.11	0.004	0.020
								**cg00743540**	0.19	0.002	0.014
*IL17RE*	8	4	cg02968508	−0.11	0.025	0.101	2				
			cg05253480	0.2	0.017	0.070					
			cg06619959	−0.16	0.003	0.016		**cg06619959**	−0.1	0.003	0.011
			cg15095327	0.13	0.043	0.151		cg15095327	0.19	0.031	0.116
*IL17REL*	12	5	**cg00692279**	0.12	4.28 × 10^−4^	0.004	4				
			cg04485799	0.14	0.026	0.101		cg04485799	0.13	0.039	0.130
			cg12009803	0.11	0.013	0.061					
			**cg26206185**	0.55	2.3 × 10^−5^	3.5 × 10^−5^					
			**cg27068297**	−0.14	0.003	0.016					
								cg00090674	0.23	**2.16 × 10^−4^**	0.002
								cg13563334	0.15	**1.96 × 10^−5^**	3.5 × 10^−5^
								cg00692279	0.13	**0.007**	0.032
*IL21*	3	1	cg00136405	−0.16	0.014	0.062	1	cg00136405	−0.16	**0.004**	0.020
*IL21R*	12	7	**cg02656594**	−0.53	2.86 × 10^−14^	<1.0 × 10^−6^	7	cg02656594	−0.52	**2.89 × 10^−15^**	<1.0 × 10^−6^
			**cg00050618**	0.26	0.001	0.006		cg00050618	0.22	**3.05 × 10^−6^**	6.0 × 10^−6^
			**cg05814654**	−0.86	5.46 × 10^−6^	1.2 × 10^−5^		cg05814654	−0.82	**1.01 × 10^−4^**	0.002
			**cg02983090**	−0.72	9.87 × 10^−6^	1.7 × 10^−5^		cg02983090	−0.73	**2.14 × 10^−7^**	1.0 × 10^−6^
			**cg08282819**	−1.07	5.33 × 10^−9^	<1.0 × 10^−6^		cg08282819	−0.96	**4.29 × 10^−14^**	<1.0 × 10^−6^
			cg19423311	−0.48	0.048	0.15494		cg19423311	−0.41	0.035	0.125
			**cg27027151**	−0.47	4.7 × 10^−11^	<1.0 × 10^−6^					
*IL22*	8	0					1	cg13851647	−0.22	0.046	0.149
*IL22RA1*	4	1	**cg21293216**	0.28	0.009	0.050	3	cg21293216	0.4	**1.55 × 10^−4^**	0.002
								cg09152089	0.18	0.032	0.116
								cg11651446	0.17	**0.002**	0.014
*IL22RA2*	2	1	cg00415333	0.14	0.050	0.155	0				
Total number	106	34					33				

^a^ CpGs with statistically significant differences after applying false discovery tests are bolded; ^b^ Changes in methylation from age 18 years to first half of pregnancy (8–21 weeks) for Treg pathway gene CpGs; **^c^** Changes in methylation from age 18 years to second half of pregnancy (22–38 weeks) for Treg pathway gene CpGs.

**Table 5:** Treg CpGs showing statistically significant changes (*p ≤* 0.05) during first and second halves of pregnancy ^a^.

Treg Gene Names	Total Number of CpGs	Significant CpGs in First Half of Pregnancy ^b^					Significant CpGs in Second Half of Pregnancy ^c^				
		No. of Sign. CpGs	CpG Sites	Parameter Estimates	*p*-Value	FDR-Adjusted *p*-Value	No. of Sign. CpGs	CpG Sites	Parameter Estimates	*p*-Value	FDR-Adjusted *p*-Value
*FOXP3*	7	3	cg01905377	−1.01	2.19 × 10^−8^	**<1.0 × 10^−6^**	4	cg01905377	−1.28	7.46 × 10^−11^	**<1.0 × 10^−6^**
			cg06767008	0.42	7.46 × 10^−12^	**<1.0 × 10^−6^**		cg06767008	0.37	2.22 × 10^−6^	0.10
			cg15614573	−0.41	0.033	0.11		cg15614573	−0.81	0.001	**1.0 × 10^−5^**
								cg04920616	0.21	0.040	**0.004**
*CTLA4*	3	0					0				
Total	10	3					4				

^a^ CpGs with statistically significant differences after applying false discovery tests are bolded; ^b^ Changes in methylation from age 18 years to first half of pregnancy (8–21 weeks) for Treg pathway gene CpGs; **^c^** Changes in methylation from age 18 years to second half of pregnancy (22–38 weeks) for Treg pathway gene CpGs.

**Table 6:** Relative risk of significant CpG changes in immune pathways in pregnancy compared to random CpGs.

Time	First Half of Pregnancy						Second Half of Pregnancy					
	Association Based on Original *p*-Value			Association Based on FDR-Tested *p*-Value			Association Based on Original *p*-Value			Association Based on FDR-Tested *p*-Value		
Pathway (Total Number of CpGs)	Proportion of Significant CpGs	Risk Ratio	95% Confidence Interval	Proportion of Significant CpGs	Risk Ratio	95% Confidence Interval	Proportion of Significant CpGs	Risk Ratio	95% Confidence Interval	Proportion of Significant CpGs	Risk Ratio	95% Confidence Interval
Compared to 10 random subsets based on the whole genome												
Th1 (155)	27.7	1.15	0.84–1.57	18.7	1.45	0.95–2.22	36.1	1.43	1.08–1.88	25.2	1.68	1.16–2.44
Th2 (77)	35.1	1.45	1.02–2.07	20.9	1.61	0.96–2.69	33.8	1.33	0.93–1.91	26.0	1.74	1.11–2.73
Th17 (106)	32.1	1.33	0.95–1.85	18.9	1.46	0.90–2.36	31.1	1.23	0.88–1.72	22.6	1.52	0.98–2.33
Treg (10)	30.0	1.24	0.47–3.26	20.0	1.55	0.44–5.51	40.0	1.58	0.72–3.44	30.0	2.01	0.75–5.35
Random (Median proportion)	24.1			12.9			24.6			14.9		
Compared to 10 random subsets based on the genes expressed in CD4+ cells												
Th1 (155)	27.7	1.16	0.84–1.59	18.7	1.30	0.86–1.97	36.1	1.35	1.03–1.78	25.2	1.72	1.18–2.49
Th2 (77)	35.1	1.46	1.02–2.09	20.9	1.45	0.87–2.40	33.8	1.26	0.88–1.81	26.0	1.77	1.13–2.79
Th17 (106)	32.1	1.34	0.96–1.87	18.9	1.31	0.82–2.10	31.1	1.17	0.84–1.62	22.6	1.55	1.00–2.38
Treg (10)	30.0	1.25	0.48–3.28	20.0	1.39	0.39–4.94	40.0	1.50	0.69–3.26	30.0	2.05	0.77–5.46
Random (Median proportion)	24.0			10.4			26.7			9.80		

The table shows the median risk ratios of significant changes of CpGs between age 18 years and pregnancy (22–24 years) in different immune pathways (Th1, Th2, Th17, Treg) compared to two sets of random samples of CpGs separate for the first and second halves of pregnancy.

**Table 7:** Number and proportion of CpG sites according to their position on the gene.

Position of CpGs on Gene ^a^	Th1, Th2, Th17 and Treg Gene CpGs (Total 348) *n* (%)	Random CpGs from Whole Genome (Total 3480) *n* (%)	Random CpGs from Genes Expressed in CD4+ Cells (Total 3480) *n* (%)	Random CpGs from Whole Genome Excluding CpGs Situated in Intergenic Region (Total 348) *n* (%)
Body, 3′UTR, and Promoter	0 (0)	1 (0.03)	0 (0)	1 (0.03)
Body, 5′UTR, and Promoter	6 (1.7)	33 (1.0)	74 (2.1)	37 (1.1)
Body and 3′UTR	3 (0.9)	23 (0.7)	26 (0.8)	34 (1.0)
Body and 5′UTR	21 (6.0)	148 (4.3)	197 (5.7)	180 (5.2)
Body and Promoter	4 (1.2)	134 (3.9)	293 (8.4)	161 (4.6)
Body	129 (37.1)	1140 (32.8)	1375 (39.5)	1489 (42.8)
3′UTR and Promoter	1 (0.3)	5 (0.1)	15 (0.4)	9 (0.1)
5′UTR and Promoter	8 (2.3)	55 (1.6)	116 (3.3)	65 (1.9)
Promoter	117 (33.6)	854 (24.5)	1005 (28.9)	1114 (32.0)
3′UTR and 5′UTR	0 (0)	0 (0)	1 (0.03)	0 (0)
3′UTR	26 (7.5)	124 (3.6)	149 (4.1)	153 (4.4)
5′UTR	33 (9.5)	200 (5.7)	229 (6.6)	237 (6.8)
Intergenic	0 (0)	763 (21.9)	0 (0)	0 (0)

^a^ Body = Body and/or 1st Exon; Promoter = TSS (transcription start site).

## Supplementary Materials

Supplementary materials can be found at http://www.mdpi.com/1422-0067/19/2/477/s1.
