# Supplementary material for: Changes in DNA Methylation from Age 18 to Pregnancy in Type 1, 2, and 17 T Helper and Regulatory T-Cells Pathway Genes
Source: Int J Mol Sci. 2018 Feb 6;19(2):477. doi: 10.3390/ijms19020477 (PMC5855699; doi:10.3390/ijms19020477)
Supplement: Supplementary file 1 [file ijms-19-00477-s001.zip › supplementary/Supplementary tables.docx]

Article

**Changes in DNA Methylation from Age 18 to Pregnancy in Type 1, 2, and 17 T Helper and Regulatory T-Cells Pathway Genes**

Sabrina Iqbal ^1^, Gabrielle A. Lockett ^2^, John W. Holloway ^2,3^, S. Hasan Arshad ^3,4^, Hongmei Zhang ^1^, Akhilesh Kaushal ^1^, Sabarinath R. Tetali ^1^, Nandini Mukherjee ^1^ and Wilfried J. J. Karmaus ^1,^*

Table 1. Participants’ characteristics with available methylation data comparing to all female participants of the cohort and comparing participants in the first and second halves of pregnancy.

| **Factor at 18 years** | **Female participants with DNA-M data at age 18 (*n* = 245)  *n* (%)** | **The pregnant women when they were 18 years of age  (*n* = 54) *n* (%)** | ***p*-value (*n* = 54 vs. *n* = 245)** | **First half of pregnancy subset (*n* = 39)  *n* (%)** | **Second half of pregnancy subset (*n* = 35)  *n* (%)** | ***p*-value (*n* = 39 vs. *n* = 35)** |
| --- | --- | --- | --- | --- | --- | --- |
| **Asthma** | | | | | | |
| No | 210 (85.7) | 44 (81.5) | 0.431 | 32 (82.1) | 28 (80.0) | 0.82 |
| Yes | 35 (14.3) | 10 (18.5) |  | 7 (17.9) | 7 (20.0) |  |
| **Eczema** | | | | | | |
| No | 207 (84.8) | 42 (77.8) | 0.205 | 35 (89.7) | 25 (74.3) | 0.07 |
| Yes | 37 (15.2) | 12 (22.2) |  | 4 (10.3) | 9 (25.7) |  |
| **Atopy** | | | | | | |
| No | 166 (68.6) | 42 (77.8) | 0.182 | 29 (74.4) | 26 (74.3) | 0.99 |
| Yes | 76 (31.4) | 12 (22.2) |  | 10 (25.6) | 9 (25.7) |  |
| **Active smoking** | | | | | | |
| No | 181 (74.2) | 33 (61.1) | 0.053 | 26 (66.7) | 19 (54.2) | 0.23 |
| Yes | 63 (25.8) | 21 (38.9) |  | 13 (33.3) | 16 (45.7) |  |
| **Multivitamin use during pregnancy** | | | | | | |
| No | - | - |  | 34 (87.2) | 31 (79.6) | - |
| Yes | - | - |  | 5 (12.8) | 4 (11.4) | - |
| **Folic Acid use during pregnancy** | | | | | | |
| No |  |  |  | 7 (17.9) | 7 (20.0) | - |
| Yes |  |  |  | 32 (82.1) | 28 (80.0) | - |
|  | **Median (5–95% values)** | **Median (5–95% values)** |  | **Median (5–95% values)** | **Median (5–95% values)** |  |
| Age of the mother | (All female participants were about 18 years of age.) | |  | 22.8 (18.9–25.3) | 22.9 (19–24.9) | n.s. |
|  |  |  |  |  |  |  |
| Body mass index of the mother * | 22.9 (19.1–32.9) | 24.9 (19.1–32.9) | n.s. | 27.9 (23.2–41.0) |  |  |

* Body mass index = weight in kg/ (height in m)^2^; n.s. = not statistically significant, tested with the non-parametric Kruskal-Wallis Test.

**Table 2.** Smoking status at age 18 years (No/Yes); for women analyzed in the first and second halves of pregnancy (never, early, transient, or smoking throughout pregnancy) ^a^.

| Pregnancy | **Age 18 (First half of Pregnancy)** | | | | **Age 18 (Second half of Pregnancy)** | | |
| --- | --- | --- | --- | --- | --- | --- | --- |
|  | **Smoking category** | **No  *n* (%)** | **Yes *n* (%)** | **Total** | **No *n* (%)** | **Yes *n* (%)** | **Total** |
|  | Never | 21 (84.00) | 5 (41.67) | 26 | 15 (78.95) | 7 (46.67) | 22 |
|  | Early | 3 (12.00) | 1 (8.33) | 4 | 3 (15.79) | 1 (6.67) | 4 |
|  | Transient | 0 (0.00) | 5 (41.67) | 5 | 1 (5.26) | 4 (26.67) | 5 |
|  | Throughoutpregnancy | 1 (4.00) | 1 (8.33) | 2 | 0 (0.00) | 3 (20.00) | 3 |
|  | Total | 25 | 12 | 37 | 19 | 15 | 34 |

^a^ Smoking information was missing for two participants in first half of pregnancy and for one participant in the second half of pregnancy.

**Table 3.** Comparison of the average estimated cell proportions between the non-pregnant state, and the first and second halves of pregnancy

|  | **Age 18 (*n* = 39)** | | | | | **First half of Pregnancy (*n* = 39)** | | | |
| --- | --- | --- | --- | --- | --- | --- | --- | --- | --- |
| **Cell Types** | **Median** | **5^th^ Percentile** | **95^th^ Percentile** | **Median** | | | | **5^th^ Percentile** | **95^th^ Percentile** |
| B cell | 0.041 | 0.005 | 0.088 | | 0.026 | | <0.001 | | 0.055 |
| CD4T cell | 0.124 | 0.030 | 0.213 | | 0.079 | | 0.038 | | 0.127 |
| CD8T cell | 0.077 | 0.035 | 0.218 | | 0.047 | | 0.013 | | 0.100 |
| Granulocytes | 0.559 | 0.300 | 0.654 | | 0.730 | | 0.624 | | 0.822 |
| Monocytes | 0.071 | 0.041 | 0.094 | | 0.072 | | 0.050 | | 0.105 |
| NK cells | 0.095 | 0.020 | 0.287 | | 0.030 | | <0.001 | | 0.068 |
| Eosinophils | 0.018 | 0 | 0.085 | | 0.004 | | 0 | | 0.044 |
|  | **Age 18 (*n* = 35)** | | | | **Second half of Pregnancy (*n* = 35)** | | | | |
| **Cell Types** | **Median** | **5^th^ Percentile** | **95^th^ Percentile** | | **Median** | | **5^th^ Percentile** | | **95^th^ Percentile** |
| B cell | 0.044 | 0.006 | 0.085 | | 0.025 | | 0.007 | | 0.056 |
| CD4T cell | 0.116 | 0.060 | 0.216 | | 0.078 | | 0.037 | | 0.135 |
| CD8T cell | 0.077 | 0.035 | 0.218 | | 0.047 | | 0.025 | | 0.107 |
| Granulocytes | 0.579 | 0.336 | 0.696 | | 0.738 | | 0.624 | | 0.822 |
| Monocytes | 0.071 | 0.042 | 0.094 | | 0.074 | | 0.051 | | 0.117 |
| NK cells | 0.079 | 0.001 | 0.258 | | 0.018 | | 0 | | 0.063 |
| Eosinophils | 0.026 | 0 | 0.086 | | 0.010 | | 0 | | 0.025 |

**Table 4.** 348 CpGs from Th1, Th2, Th17 and Treg pathway genes showing changes from age 18 years to both first and second halves of pregnancy.

| **source** | **Gene name** | **CpGs** | **UCSC_RefGene_Group** | **Chromo-some number** | **First half of pregnancy** | | | **Second half of pregnancy** | | |
| --- | --- | --- | --- | --- | --- | --- | --- | --- | --- | --- |
|  |  |  |  |  | **Estimate** | ***p*-value** | **FDR *p*-value** | **Estimate** | ***p*-value** | **FDR *p*-value** |
| Th1 | *IFNG* | cg01940810 | Body | 12 | 0.15 | 0.033538 | 0.14528 | 0.12 | 0.207654 | 0.397 |
| Th1 | *IFNG* | cg05224770 | TSS1500 | 12 | 0.14 | 0.062170 | 0.1927 | 0.16 | 0.050788 | 0.135 |
| Th1 | *IFNG* | cg12640631 | TSS1500 | 12 | −0.06 | 0.692054 | 0.864347 | −0.12 | 0.356489 | 0.553 |
| Th1 | *IFNG* | cg26227465 | TSS200 | 12 | 0.1 | 0.10721 | 0.283808 | 0.13 | 0.014495 | 0.056 |
| Th1 | *IFNG*; *IFNG* | cg00848007 | 1stExon; 5′UTR | 12 | 0.02 | 0.842775 | 0.91993 | −0.09 | 0.482824 | 0.640 |
| Th1 | *IFNGR1* | cg07401792 | Body | 6 | 0.05 | 0.730462 | 0.864347 | −0.25 | 0.011146 | 0.044 |
| Th1 | *IFNGR1* | cg14279899 | TSS1500 | 6 | −0.16 | 0.087307 | 0.237415 | −0.02 | 0.832963 | 0.911 |
| Th1 | *IFNGR1* | cg26668632 | TSS1500 | 6 | −0.14 | 0.045397 | 0.163641 | −0.11 | 0.051415 | 0.135 |
| Th1 | *IFNGR2* | cg07845895 | Body | 21 | −0.2 | 0.176254 | 0.395933 | −0.14 | 0.190732 | 0.379 |
| Th1 | *IFNGR2* | cg08173915 | TSS1500 | 21 | −0.49 | <1.1E−19 | 1.46E−08 | −0.44 | <1.1E−19 | 1.5E−08 |
| Th1 | *IFNGR2* | cg08874470 | Body | 21 | −0.07 | 0.050162 | 0.176706 | 0.04 | 0.431414 | 0.609 |
| Th1 | *IFNGR2* | cg10572943 | TSS200 | 21 | 0.14 | 0.111692 | 0.283808 | 0.19 | 0.079904 | 0.197 |
| Th1 | *IFNGR2* | cg17356733 | TSS1500 | 21 | −0.5 | 4.44E−16 | 6.88E−14 | −0.43 | 3.48E−11 | 1.1E−09 |
| Th1 | *IFNGR2* | cg20817150 | Body | 21 | 0.03 | 0.680374 | 0.864347 | 0.04 | 0.441646 | 0.609 |
| Th1 | *IFNGR2* | cg21664037 | 3′UTR | 21 | −0.04 | 0.707135 | 0.864347 | 0.1 | 0.384117 | 0.578 |
| Th1 | *IFNGR2* | cg22669060 | TSS1500 | 21 | −0.49 | 1.01E−12 | 6.95E−11 | −0.47 | 1.05E−10 | 2.7E−09 |
| Th1 | *IFNGR2* | cg24865779 | TSS200 | 21 | −0.09 | 0.429600 | 0.697404 | −0.05 | 0.557673 | 0.714 |
| Th1 | *IFNGR2* | cg27469991 | Body | 21 | 0.03 | 0.642542 | 0.864347 | −0.15 | 0.018271 | 0.066 |
| Th1 | *IL12A* | cg08017498 | TSS200 | 3 | 0 | 0.984991 | 0.989348 | 0.04 | 0.604108 | 0.752 |
| Th1 | *IL12A* | cg09362366 | Body | 3 | −0.56 | 0.00066 | 0.004091 | 0.1 | 0.43185 | 0.609 |
| Th1 | *IL12A* | cg14995416 | 3′UTR | 3 | −0.1 | 0.247999 | 0.460700 | −0.06 | 0.517877 | 0.680 |
| Th1 | *IL12A* | cg20515136 | Body | 3 | −0.31 | 4.34E−06 | 4.87E−05 | −0.5 | 1.16E−07 | 1.4E−06 |
| Th1 | *IL12A* | cg20524905 | TSS1500 | 3 | −0.06 | 0.404399 | 0.675230 | −0.12 | 0.157652 | 0.317 |
| Th1 | *IL12A* | cg25777557 | TSS200 | 3 | 0.18 | 0.187043 | 0.410568 | 0.09 | 0.459241 | 0.624 |
| Th1 | *IL12A* | cg25829945 | TSS1500 | 3 | −0.24 | 0.040762 | 0.150432 | −0.18 | 0.038798 | 0.113 |
| Th1 | *IL12B* | cg00066816 | TSS1500 | 5 | −0.06 | 0.249669 | 0.460700 | 0.01 | 0.869842 | 0.932 |
| Th1 | *IL12B* | cg06111286 | TSS200 | 5 | −0.39 | 2.31E−05 | 0.000239 | −0.45 | 3.16E−09 | 4.1E−08 |
| Th1 | *IL12B* | cg12368524 | TSS1500 | 5 | −0.04 | 0.711910 | 0.864347 | −0.13 | 0.242977 | 0.443 |
| Th1 | *IL12B* | cg14587604 | TSS1500 | 5 | −0.04 | 0.706290 | 0.864347 | −0.2 | 0.122233 | 0.260 |
| Th1 | *IL12B* | cg16045612 | TSS1500 | 5 | 0.05 | 0.518328 | 0.772508 | 0.04 | 0.481105 | 0.640 |
| Th1 | *IL12B* | cg18060742 | 3′UTR | 5 | −0.11 | 0.357508 | 0.622627 | 0.08 | 0.267109 | 0.470 |
| Th1 | *IL12B* | cg21116673 | Body | 5 | 0.08 | 0.174836 | 0.395933 | 0.07 | 0.367466 | 0.559 |
| Th1 | *IL12B* | cg25093583 | TSS1500 | 5 | −0.01 | 0.891805 | 0.953309 | 0.06 | 0.093058 | 0.219 |
| Th1 | *IL12B; IL12B* | cg18307303 | 1stExon; 5′UTR | 5 | −0.09 | 0.245318 | 0.460700 | −0.14 | 0.015649 | 0.059 |
| Th1 | *IL12RB1* | cg26642774 | 3′UTR | 19 | −0.52 | 0.109435 | 0.283808 | −0.33 | 0.464518 | 0.626 |
| Th1 | *IL12RB1; IL12RB1* | cg09365686 | Body; Body | 19 | −0.09 | 0.161041 | 0.390022 | −0.08 | 0.10478 | 0.235 |
| Th1 | *IL12RB1; IL12RB1* | cg10140613 | TSS1500; TSS1500 | 19 | 0.07 | 0.319705 | 0.563116 | 0.03 | 0.664543 | 0.798 |
| Th1 | *IL12RB1; IL12RB1* | cg11992736 | Body; Body | 19 | −0.02 | 0.816197 | 0.897237 | 0.1 | 0.280791 | 0.489 |
| Th1 | *IL12RB1; IL12RB1* | cg12123019 | TSS200; TSS200 | 19 | −0.5 | 0.000134 | 0.001100 | −0.33 | 0.000696 | 0.005 |
| Th1 | *IL12RB1; IL12RB1* | cg24755266 | Body; 3′UTR | 19 | 0.13 | 0.15120 | 0.372024 | −0.02 | 0.834453 | 0.911 |
| Th1 | *IL12RB2* | cg02566391 | Body | 1 | 0.46 | 1.48E−12 | 6.95E−11 | 0.49 | <1.1E−19 | 1.6E−08 |
| Th1 | *IL12RB2* | cg04311496 | 5′UTR | 1 | −0.11 | 0.247327 | 0.4607003 | 0.08 | 0.427725 | 0.609 |
| Th1 | *IL12RB2* | cg06952660 | TSS200 | 1 | −0.29 | 0.030422 | 0.1397526 | −0.32 | 0.000354 | 0.003 |
| Th1 | *IL12RB2* | cg07192622 | 5′UTR | 1 | −0.03 | 0.751956 | 0.8698000 | −0.1 | 0.345 | 0.553 |
| Th1 | *IL12RB2* | cg09018107 | Body | 1 | 0.48 | 7.67E−10 | 1.46E−08 | 0.37 | 2.4E−06 | 2.4E−05 |
| Th1 | *IL12RB2* | cg11132246 | TSS200 | 1 | 0.18 | 0.199418 | 0.4176997 | −0.27 | 0.021 | 1 |
| Th1 | *IL12RB2* | cg12633410 | 5′UTR | 1 | 0.21 | 0.030655 | 0.1397526 | 0.16 | 0.02466 | 0.0813245 |
| Th1 | *IL12RB2* | cg14849855 | Body | 1 | 0.32 | 1.53E−07 | 1.98E−06 | 0.19 | 0.003899 | 0.0208413 |
| Th1 | *IL12RB2* | cg19745415 | TSS200 | 1 | −0.49 | 0.000439 | 0.0029641 | −0.41 | 0.002786 | 0.0159965 |
| Th1 | *IL12RB2* | cg20253742 | 5′UTR | 1 | −0.53 | 7.27E−05 | 0.0006631 | −0.25 | 0.002011 | 0.0124676 |
| Th1 | *IL2* | cg25065535 | Body | 4 | 0.04 | 0.796922 | 0.8886545 | 0.03 | 0.757935 | 0.8739816 |
| Th1 | *IL2RA* | cg27131821 | TSS1500 | 10 | −0.12 | 0.051813 | 0.1776546 | −0.1 | 0.089101 | 0.2124719 |
| Th1 | *IL2RA; IL2RA* | cg26316423 | 1stExon; 5′UTR | 10 | −0.07 | 0.366311 | 0.6308693 | −0.05 | 0.367939 | 0.5591232 |
| Th1 | *IL2RB* | cg11558856 | TSS200 | 22 | 0.11 | 0.190817 | 0.4105684 | 0 | 0.978964 | 0.9789635 |
| Th1 | *IL2RB* | cg21307484 | TSS1500 | 22 | 0.27 | 6.63E−05 | 0.0006418 | 0.18 | 0.001777 | 0.0114786 |
| Th1 | *IL2RB* | cg24509815 | 3′UTR | 22 | −0.03 | 0.474867 | 0.7146060 | 0.18 | 2.13E−09 | 3.00E−08 |
| Th1 | *IL2RB* | cg26757673 | 5′UTR | 22 | 0.08 | 0.307372 | 0.5539861 | 0.23 | 4.09E−07 | 4.23E−06 |
| Th1 | *TNF* | cg01360627 | Body | 6 | 0.16 | 0.005782 | 0.0309074 | 0.3 | 2.42E−11 | 9.36E−10 |
| Th1 | *TNF* | cg04425624 | 1stExon | 6 | −0.27 | 0.001962 | 0.0108626 | −0.06 | 0.41166 | 0.5963301 |
| Th1 | *TNF* | cg04472685 | 3′UTR | 6 | 0.15 | 0.086384 | 0.2374159 | 0.1 | 0.113396 | 0.2475544 |
| Th1 | *TNF* | cg09637172 | Body | 6 | 0.02 | 0.877461 | 0.9444902 | 0.03 | 0.76121 | 0.8739816 |
| Th1 | *TNF* | cg10650821 | 1stExon | 6 | −0.33 | 0.000142 | 0.0011062 | −0.3 | 0.000745 | 0.0050228 |
| Th1 | *TNF* | cg10717214 | 1stExon | 6 | −0.25 | 0.001834 | 0.0105301 | −0.13 | 0.096408 | 0.2230332 |
| Th1 | *TNF* | cg11484872 | TSS200 | 6 | −0.31 | 0.052723 | 0.1776545 | 0 | 0.97348 | 0.9789635 |
| Th1 | *TNF* | cg15989608 | 3′UTR | 6 | 0.12 | 0.407921 | 0.6752300 | 0.21 | 0.04345 | 0.1224488 |
| Th1 | *TNF* | cg17755321 | 3′UTR | 6 | 0.1 | 0.241250 | 0.4607003 | 0.15 | 0.027192 | 0.0878085 |
| Th1 | *TNF* | cg23384708 | Body | 6 | 0.06 | 0.219965 | 0.4486134 | 0.22 | 6.21E−05 | 0.0005062 |
| Th1 | *TNF* | cg26736341 | 3′UTR | 6 | 0.12 | 0.081569 | 0.2325726 | 0.14 | 0.031909 | 0.0989182 |
| Th1 | *TNFAIP1* | cg11814826 | Body | 17 | −0.03 | 0.785576 | 0.8886541 | −0.24 | 0.010281 | 0.0430677 |
| Th1 | *TNFAIP1* | cg13290523 | Body | 17 | −0.08 | 0.314351 | 0.5600509 | −0.18 | 0.030539 | 0.0966024 |
| Th1 | *TNFAIP1* | cg15159588 | 3′UTR | 17 | −0.07 | 0.300594 | 0.5481428 | −0.11 | 0.30492 | 0.5137234 |
| Th1 | *TNFAIP1; IFT20* | cg22640868 | TSS1500; 5′UTR | 17 | −0.5 | 2.29E−12 | 7.10E−11 | −0.46 | 1.27E−13 | 6.55E−12 |
| Th1 | *TNFAIP1*; *IFT20*; *IFT20* | cg26663469 | TSS200; 5′UTR; 1stExon | 17 | −0.03 | 0.643212 | 0.8643472 | 0.16 | 0.033172 | 0.1008178 |
| Th1 | *TNFAIP2* | cg03021690 | TSS1500 | 14 | 0.12 | 0.036299 | 0.1452878 | 0.14 | 0.002453 | 0.0146208 |
| Th1 | *TNFAIP2* | cg03572388 | Body | 14 | 0.02 | 0.696888 | 0.8643472 | −0.12 | 0.049311 | 0.1350732 |
| Th1 | *TNFAIP2* | cg05288750 | TSS200 | 14 | −0.04 | 0.442922 | 0.7019738 | 0.02 | 0.664324 | 0.798482 |
| Th1 | *TNFAIP2* | cg09274347 | Body | 14 | 0.13 | 0.055653 | 0.1835391 | 0.02 | 0.72543 | 0.8583336 |
| Th1 | *TNFAIP2* | cg13144594 | Body | 14 | 0.23 | 0.011592 | 0.0579614 | 0.26 | 0.015964 | 0.0589159 |
| Th1 | *TNFAIP2* | cg16127617 | Body | 14 | −0.05 | 0.740324 | 0.8643472 | −0.15 | 0.216113 | 0.4035845 |
| Th1 | *TNFAIP2* | cg20368904 | Body | 14 | 0.01 | 0.931359 | 0.9688638 | 0.09 | 0.444115 | 0.6091843 |
| Th1 | *TNFAIP2; TNFAIP2* | cg04264002 | 5′UTR; 1stExon | 14 | 0 | 0.96039 | 0.9858307 | 0.14 | 0.006925 | 0.0325284 |
| Th1 | *TNFAIP3* | cg00847892 | TSS200 | 6 | −0.03 | 0.741665 | 0.8643472 | −0.08 | 0.392954 | 0.5800747 |
| Th1 | *TNFAIP3* | cg06779945 | TSS1500 | 6 | 0.17 | 0.039416 | 0.1490118 | 0.03 | 0.740953 | 0.8700589 |
| Th1 | *TNFAIP3* | cg08667148 | TSS200 | 6 | 0.03 | 0.679983 | 0.8643472 | 0 | 0.951274 | 0.9646115 |
| Th1 | *TNFAIP3* | cg08919597 | Body | 6 | −0.84 | <1.1E−19 | 1.46E−08 | −0.43 | 0.00321 | 0.0177707 |
| Th1 | *TNFAIP3* | cg11812071 | Body | 6 | −0.09 | 0.149967 | 0.3720243 | 0.11 | 0.288103 | 0.4961769 |
| Th1 | *TNFAIP3* | cg12200164 | Body | 6 | −0.19 | 0.034801 | 0.1452878 | −0.16 | 0.07238 | 0.1809507 |
| Th1 | *TNFAIP3* | cg12214665 | TSS200 | 6 | −0.01 | 0.861342 | 0.9336234 | −0.14 | 0.010957 | 0.0442979 |
| Th1 | *TNFAIP3* | cg15380607 | TSS200 | 6 | −0.05 | 0.409494 | 0.6752300 | −0.07 | 0.256318 | 0.4566577 |
| Th1 | *TNFAIP3* | cg18287768 | Body | 6 | 0.09 | 0.05727 | 0.184956 | 0.02 | 0.527768 | 0.6846529 |
| Th1 | *TNFAIP3* | cg19862242 | Body | 6 | 0 | 0.983549 | 0.9893483 | 0.01 | 0.882054 | 0.932471 |
| Th1 | *TNFAIP3* | cg23696891 | TSS1500 | 6 | 0.2 | 0.173195 | 0.3959330 | −0.06 | 0.613711 | 0.7549617 |
| Th1 | *TNFAIP3* | cg25971086 | 5′UTR | 6 | −0.04 | 0.565787 | 0.8288023 | 0.01 | 0.884343 | 0.932471 |
| Th1 | *TNFAIP3; TNFAIP3* | cg18264753 | 5′UTR; 1stExon | 6 | −0.13 | 0.017539 | 0.0849590 | −0.13 | 0.050437 | 0.1350732 |
| Th1 | *TNFAIP6* | cg01035238 | TSS1500 | 2 | −0.04 | 0.718811 | 0.8643472 | −0.1 | 0.349812 | 0.5525579 |
| Th1 | *TNFAIP6* | cg01189638 | TSS1500 | 2 | −0.05 | 0.572140 | 0.8288023 | 0.08 | 0.391292 | 0.5800747 |
| Th1 | *TNFAIP6* | cg01974138 | TSS1500 | 2 | −0.11 | 0.248410 | 0.4607003 | −0.1 | 0.292887 | 0.4988732 |
| Th1 | *TNFAIP6* | cg03406844 | Body | 2 | −0.49 | 1.79E−12 | 6.95E−11 | −0.51 | 4.44E−16 | 6.88E−14 |
| Th1 | *TNFAIP8* | cg00421693 | Body | 5 | 0.12 | 0.174658 | 0.3959330 | −0.07 | 0.342474 | 0.5525579 |
| Th1 | *TNFAIP8* | cg00524900 | Body | 5 | −0.71 | 1.03E−09 | 1.46E−08 | −0.88 | <1.1E−19 | 1.55E−08 |
| Th1 | *TNFAIP8* | cg01057573 | Body | 5 | −0.73 | <1.1E−19 | 1.46E−08 | −0.72 | <1.1E−19 | 1.55E−08 |
| Th1 | *TNFAIP8* | cg01915433 | Body | 5 | 0.02 | 0.733812 | 0.8643472 | −0.08 | 0.354488 | 0.5525579 |
| Th1 | *TNFAIP8* | cg03665078 | Body | 5 | −0.21 | 0.00113 | 0.0067368 | −0.17 | 0.008982 | 0.0397766 |
| Th1 | *TNFAIP8* | cg03723497 | TSS1500 | 5 | 0 | 0.989348 | 0.9893483 | 0.02 | 0.816143 | 0.910847 |
| Th1 | *TNFAIP8* | cg04280246 | TSS200 | 5 | 0.11 | 0.228427 | 0.4590916 | −0.02 | 0.826819 | 0.910847 |
| Th1 | *TNFAIP8* | cg07398791 | Body | 5 | −0.31 | 4.40E−06 | 4.87E−05 | −0.35 | 1.83E−07 | 2.03E−06 |
| Th1 | *TNFAIP8* | cg11846226 | Body | 5 | 0.22 | 0.000133 | 0.0011006 | −0.01 | 0.875721 | 0.932471 |
| Th1 | *TNFAIP8* | cg12148675 | Body | 5 | −0.18 | 0.036556 | 0.1452878 | 0.03 | 0.759635 | 0.8739816 |
| Th1 | *TNFAIP8* | cg15292768 | Body | 5 | −0.04 | 0.570540 | 0.8288023 | −0.09 | 0.153913 | 0.3139023 |
| Th1 | *TNFAIP8* | cg15408889 | Body | 5 | −0.2 | 0.010163 | 0.0525126 | −0.2 | 0.009748 | 0.0419719 |
| Th1 | *TNFAIP8* | cg18689486 | TSS1500 | 5 | −0.03 | 0.789469 | 0.8886541 | 0.02 | 0.872569 | 0.932471 |
| Th1 | *TNFAIP8* | cg21239001 | Body | 5 | −0.45 | 6.10E−12 | 1.58E−10 | −0.49 | 8.88E−16 | 6.88E−14 |
| Th1 | *TNFAIP8* | cg26305729 | Body | 5 | −0.04 | 0.591713 | 0.8492186 | −0.13 | 0.067103 | 0.1705067 |
| Th1 | *TNFAIP8; TNFAIP8* | cg02283238 | Body; TSS1500 | 5 | −0.08 | 0.444753 | 0.7019738 | 0.12 | 0.197886 | 0.3873457 |
| Th1 | *TNFAIP8; TNFAIP8* | cg07086380 | Body; TSS1500 | 5 | 0.22 | 0.000267 | 0.0018846 | 0.27 | 6.82E−05 | 0.0005289 |
| Th1 | *TNFAIP8; TNFAIP8* | cg14692284 | Body; Body | 5 | −0.06 | 0.383375 | 0.6530026 | −0.1 | 0.084865 | 0.2055328 |
| Th1 | *TNFAIP8; TNFAIP8* | cg16645133 | Body; TSS200 | 5 | −0.06 | 0.673274 | 0.8643472 | 0.2 | 0.106568 | 0.2359727 |
| Th1 | *TNFAIP8; TNFAIP8* | cg17008454 | Body; Body | 5 | 0.17 | 0.077561 | 0.2282012 | 0.06 | 0.353549 | 0.5525579 |
| Th1 | *TNFAIP8; TNFAIP8* | cg24120357 | Body; TSS200 | 5 | 0.07 | 0.471453 | 0.7146060 | 0.01 | 0.945669 | 0.9646115 |
| Th1 | *TNFAIP8; TNFAIP8* | cg24957532 | 3′UTR; 3′UTR | 5 | 0.01 | 0.924749 | 0.9688623 | −0.14 | 0.19992 | 0.3873457 |
| Th1 | *TNFAIP8L1; TNFAIP8L1* | cg00356811 | TSS1500; TSS1500 | 19 | −0.01 | 0.925107 | 0.9688623 | −0.02 | 0.832776 | 0.910847 |
| Th1 | *TNFAIP8L1; TNFAIP8L1* | cg02436098 | 1stExon; 5′UTR; 5′UTR | 19 | −0.13 | 0.231026 | 0.4590916 | −0.2 | 0.006506 | 0.0315123 |
| Th1 | *TNFAIP8L1; TNFAIP8L1* | cg03032253 | 3′UTR; 3′UTR | 19 | 0.19 | 0.076459 | 0.2282012 | 0.07 | 0.308576 | 0.5142929 |
| Th1 | *TNFAIP8L1; TNFAIP8L1* | cg03694433 | 5′UTR; 5′UTR | 19 | 0.02 | 0.734579 | 0.8643472 | −0.07 | 0.129918 | 0.2684963 |
| Th1 | *TNFAIP8L1; TNFAIP8L1* | cg06396324 | 3′UTR; 3′UTR | 19 | 0.02 | 0.696592 | 0.8643472 | 0.03 | 0.603526 | 0.7517615 |
| Th1 | *TNFAIP8L1; TNFAIP8L1* | cg11708963 | TSS1500; TSS200 | 19 | 0 | 0.956586 | 0.9858307 | −0.17 | 0.023036 | 0.0793472 |
| Th1 | *TNFAIP8L1; TNFAIP8L1* | cg13357752 | TSS1500; TSS200 | 19 | 0.03 | 0.6699563 | 0.86434723 | −0.05 | 0.240985 | 0.4430766 |
| Th1 | *TNFAIP8L1; TNFAIP8L1* | cg22754389 | Body; Body | 19 | 0.1 | 0.1115097 | 0.2838086 | −0.15 | 0.036185 | 0.1078584 |
| Th1 | *TNFAIP8L1; TNFAIP8L1* | cg25504222 | TSS1500; TSS200 | 19 | −0.13 | 0.1747556 | 0.39593302 | 0.01 | 0.897192 | 0.9352905 |
| Th1 | *TNFAIP8L2* | cg00318000 | TSS1500 | 1 | 0.1 | 0.2056818 | 0.42507566 | −0.04 | 0.590135 | 0.7497622 |
| Th1 | *TNFAIP8L2* | cg16565154 | TSS200 | 1 | 0.11 | 0.460346 | 0.70647156 | −0.01 | 0.899086 | 0.9352905 |
| Th1 | *TNFAIP8L2* | cg23612220 | 5′UTR | 1 | −0.37 | 0.0780301 | 0.22820123 | −0.44 | 0.004967 | 0.0256647 |
| Th1 | *TNFAIP8L2; TNFAIP8L2* | cg21544402 | 1stExon; 5′UTR | 1 | −0.33 | 0.0004615 | 0.00298052 | −0.09 | 0.402894 | 0.589138 |
| Th1 | *TNFAIP8L3* | cg00063471 | Body | 15 | −0.05 | 0.7041163 | 0.86434723 | −0.06 | 0.530054 | 0.6846529 |
| Th1 | *TNFAIP8L3* | cg01058070 | TSS1500 | 15 | 0.01 | 0.8124279 | 0.89723783 | 0 | 0.952165 | 0.9646115 |
| Th1 | *TNFAIP8L3* | cg02233197 | Body | 15 | 0.06 | 0.4319411 | 0.69740486 | 0.18 | 0.008283 | 0.0377626 |
| Th1 | *TNFAIP8L3* | cg02346713 | Body | 15 | −0.16 | 0.0825258 | 0.23257265 | −0.25 | 1.37E−05 | 0.0001247 |
| Th1 | *TNFAIP8L3* | cg03454639 | Body | 15 | 0.19 | 0.0612162 | 0.19272735 | 0.22 | 0.024255 | 0.0813245 |
| Th1 | *TNFAIP8L3* | cg04659317 | Body | 15 | −0.03 | 0.6983506 | 0.86434723 | 0.04 | 0.618663 | 0.7550609 |
| Th1 | *TNFAIP8L3* | cg05503460 | Body | 15 | −0.26 | 0.0002156 | 0.00159146 | −0.3 | 3.25E−05 | 0.00028 |
| Th1 | *TNFAIP8L3* | cg06813578 | Body | 15 | 0.04 | 0.4586193 | 0.70647156 | −0.01 | 0.820211 | 0.910847 |
| Th1 | *TNFAIP8L3* | cg07218022 | Body | 15 | 0.05 | 0.670127 | 0.86434723 | −0.09 | 0.341461 | 0.5525579 |
| Th1 | *TNFAIP8L3* | cg12122631 | Body | 15 | −0.15 | 0.0386683 | 0.14901181 | 0.02 | 0.776071 | 0.884493 |
| Th1 | *TNFAIP8L3* | cg17180386 | Body | 15 | 0.02 | 0.772998 | 0.88751621 | −0.05 | 0.211159 | 0.3991429 |
| Th1 | *TNFAIP8L3* | cg19592671 | Body | 15 | 0.03 | 0.792498 | 0.88865415 | 0.15 | 0.09797 | 0.2233151 |
| Th1 | *TNFAIP8L3* | cg20318580 | TSS1500 | 15 | 0 | 0.974847 | 0.98934837 | 0.01 | 0.938667 | 0.9646115 |
| Th1 | *TNFAIP8L3* | cg22038124 | Body | 15 | 0.03 | 0.7342494 | 0.86434723 | 0.1 | 0.039235 | 0.1126185 |
| Th1 | *TNFAIP8L3* | cg23343680 | 1stExon | 15 | −0.15 | 0.0338593 | 0.14528788 | −0.15 | 0.005781 | 0.0289073 |
| Th1 | *TNFAIP8L3* | cg24034459 | 3′UTR | 15 | 0.03 | 0.699149 | 0.86434723 | −0.07 | 0.443615 | 0.6091843 |
| Th1 | *TNFAIP8L3* | cg27017085 | Body | 15 | 0.08 | 0.1910976 | 0.4105684 | −0.02 | 0.695944 | 0.8297797 |
| Th2 | *GATA3; GATA3* | cg00463367 | Body; Body | 10 | 0.18 | 0.024928 | 0.09597276 | 0.29 | 2.94E−06 | 1.88E−05 |
| Th2 | *GATA3; GATA3* | cg01255894 | Body; Body | 10 | −0.27 | 0.0136434 | 0.0552918 | −0.11 | 0.225844 | 0.4347503 |
| Th2 | *GATA3; GATA3* | cg03669298 | Body; Body | 10 | −0.32 | 0.0001021 | 0.0008738 | −0.28 | 0.000239 | 0.0014176 |
| Th2 | *GATA3; GATA3* | cg04213746 | Body; Body | 10 | 0.09 | 0.4135971 | 0.56869602 | −0.08 | 0.509618 | 0.6884311 |
| Th2 | *GATA3; GATA3* | cg04492228 | Body; Body | 10 | 0.06 | 0.5434549 | 0.64833863 | −0.07 | 0.36519 | 0.5623921 |
| Th2 | *GATA3; GATA3* | cg07989490 | 3′UTR; 3′UTR | 10 | 0.02 | 0.8558299 | 0.89052571 | −0.03 | 0.731676 | 0.8635315 |
| Th2 | *GATA3; GATA3* | cg10008757 | 5′UTR; 5′UTR | 10 | −0.15 | 0.0071269 | 0.03429832 | −0.2 | 0.005515 | 0.0265388 |
| Th2 | *GATA3; GATA3* | cg10089865 | Body; Body | 10 | 0.2 | 0.0641312 | 0.17027938 | 0.12 | 0.289269 | 0.5062199 |
| Th2 | *GATA3; GATA3* | cg11430077 | Body; Body | 10 | 0.38 | 0.0007155 | 0.0045911 | 0.18 | 0.215048 | 0.4347503 |
| Th2 | *GATA3; GATA3* | cg12181459 | Body; Body | 10 | 0.12 | 0.1619639 | 0.34642284 | −0.04 | 0.692117 | 0.8459211 |
| Th2 | *GATA3; GATA3* | cg14327531 | 5′UTR; 5′UTR | 10 | 0.07 | 0.3965287 | 0.56869602 | 0.09 | 0.299002 | 0.5102828 |
| Th2 | *GATA3; GATA3* | cg17124583 | Body; Body | 10 | 0.33 | 0.0523906 | 0.14407415 | 0.4 | 0.050684 | 0.1445445 |
| Th2 | *GATA3; GATA3* | cg17489908 | Body; Body | 10 | −0.04 | 0.6483406 | 0.73415036 | −0.07 | 0.382224 | 0.565986 |
| Th2 | *GATA3; GATA3* | cg18599069 | 5′UTR; 5′UTR | 10 | 0.1 | 0.1147566 | 0.26776539 | 0.09 | 0.118702 | 0.2991874 |
| Th2 | *GATA3; GATA3* | cg19883813 | Body; Body | 10 | 0.11 | 0.371042 | 0.56669173 | 0.12 | 0.321412 | 0.5265685 |
| Th2 | *GATA3; GATA3* | cg22770911 | Body; Body | 10 | 0.24 | 0.0002272 | 0.00174962 | 0.12 | 0.021088 | 0.0738087 |
| Th2 | *GATA3; GATA3* | cg27409129 | Body; Body | 10 | 0 | 0.9515955 | 0.95159551 | −0.1 | 0.135241 | 0.3155614 |
| Th2 | *IL13* | cg11798521 | 3′UTR | 5 | −0.03 | 0.5183344 | 0.64833863 | −0.01 | 0.847329 | 0.9455696 |
| Th2 | *IL13* | cg13566430 | TSS1500 | 5 | −0.08 | 0.2571068 | 0.43993838 | −0.13 | 0.042311 | 0.130317 |
| Th2 | *IL13* | cg15329179 | TSS200 | 5 | 0.19 | 0.0047471 | 0.02436825 | 0.01 | 0.921908 | 0.9727356 |
| Th2 | *IL13* | cg24580593 | Body | 5 | −0.03 | 0.6661744 | 0.74050819 | 0.05 | 0.341025 | 0.5358969 |
| Th2 | *IL13RA1* | cg01080862 | TSS200 | 26 | 1.8 | 1.27E−09 | 1.63E−08 | 1.77 | 1.41E−12 | 1.09E−10 |
| Th2 | *IL13RA1* | cg08750061 | TSS1500 | 26 | 0.05 | 0.4870777 | 0.63567769 | 0 | 0.973972 | 0.995332 |
| Th2 | *IL13RA1* | cg22817042 | TSS200 | 26 | 1.19 | 6.81E−10 | 1.31E−08 | 0.94 | 7.48E−10 | 1.10E−08 |
| Th2 | *IL13RA1* | cg23508470 | Body | 26 | −0.13 | 0.0390847 | 0.1262316 | −0.16 | 0.011618 | 0.0470829 |
| Th2 | *IL13RA1* | cg25968748 | Body | 26 | 1.06 | 9.49E−13 | 2.44E−11 | 0.76 | 3.99E−08 | 3.84E−07 |
| Th2 | *IL13RA1* | cg27501007 | TSS200 | 26 | 1.92 | <1.1E−19 | 1.54E−08 | 1.76 | <1.1E−19 | 1.10E−08 |
| Th2 | *IL13RA2* | cg02998206 | 5′UTR | 26 | −0.16 | 0.1586082 | 0.34642284 | −0.09 | 0.235247 | 0.4418052 |
| Th2 | *IL13RA2* | cg03244736 | TSS1500 | 26 | −0.93 | 0.0008128 | 0.00481414 | −0.53 | 0.012803 | 0.0492931 |
| Th2 | *IL1RL1* | cg11916609 | TSS1500 | 2 | −0.35 | 0.0024675 | 0.0135715 | −0.36 | 6.14E−07 | 4.30E−06 |
| Th2 | *IL1RL1* | cg16386158 | TSS1500 | 2 | 0.18 | 0.0743989 | 0.1847973 | 0.05 | 0.462451 | 0.659421 |
| Th2 | *IL1RL1* | cg17738684 | TSS1500 | 2 | 0.15 | 0.0483577 | 0.13790892 | 0.12 | 0.120452 | 0.2991874 |
| Th2 | *IL1RL1; IL1RL1* | cg12076546 | 3′UTR; Body | 2 | 0.07 | 0.5348879 | 0.64833863 | 0.02 | 0.823103 | 0.9320428 |
| Th2 | *IL4; IL4* | cg23943829 | TSS1500; TSS1500 | 5 | 0.06 | 0.3051155 | 0.47946724 | 0.05 | 0.165777 | 0.3647101 |
| Th2 | *IL4R* | cg01165142 | Body | 16 | −0.34 | 1.63E−06 | 1.80E−05 | −0.39 | 9.78E−08 | 7.53E−07 |
| Th2 | *IL4R* | cg05903710 | 3′UTR | 16 | −0.14 | 0.042687 | 0.1264192 | 0.01 | 0.922204 | 0.9727356 |
| Th2 | *IL4R; IL4R* | cg05729093 | TSS1500; TSS1500 | 16 | −0.04 | 0.6022008 | 0.69208148 | 0.21 | 0.00819 | 0.0350365 |
| Th2 | *IL4R; IL4R* | cg08317580 | 5′UTR; 5′UTR | 16 | 0.1 | 0.3014097 | 0.47946724 | −0.15 | 0.267748 | 0.4908712 |
| Th2 | *IL4R; IL4R* | cg08932316 | TSS1500; TSS1500 | 16 | −0.07 | 0.4103722 | 0.56869602 | 0 | 0.970773 | 0.995332 |
| Th2 | *IL4R; IL4R* | cg09791102 | Body; Body | 16 | −0.05 | 0.5616084 | 0.65520977 | 0.13 | 0.130974 | 0.3151556 |
| Th2 | *IL4R; IL4R* | cg16649560 | 5′UTR; 5′UTR | 16 | −0.54 | 5.50E−13 | 2.12E−11 | −0.53 | <1.1E−19 | 1.10E−08 |
| Th2 | *IL4R; IL4R* | cg26937798 | 5′UTR; 5′UTR | 16 | −0.32 | 0.0422196 | 0.1264192 | −0.3 | 0.033439 | 0.1119466 |
| Th2 | *IL5* | cg26081812 | TSS1500 | 5 | −0.02 | 0.8244822 | 0.87030415 | −0.05 | 0.501708 | 0.6884311 |
| Th2 | *IL5; IL5* | cg16184131 | 5′UTR; 1stExon | 5 | −0.03 | 0.7722921 | 0.83755627 | −0.39 | 4.52E−08 | 3.87E−07 |
| Th2 | *IL5RA; IL5RA* | cg23828301 | Body; Body | 3 | −0.68 | 2.64E−13 | 2.04E−11 | −0.67 | <1.1E−19 | 1.10E−08 |
| Th2 | *IL5RA; IL5RA;* | cg01310029 | TSS1500; TSS1500; | 3 | 0.19 | 0.0344204 | 0.12047153 | 0.03 | 0.74017 | 0.8635315 |
| Th2 | *IL5RA; IL5RA; IL5RA* | cg08404225 | 5′UTR; 5′UTR; 1stExon; 5′ | 3 | 0.13 | 0.197131 | 0.38920732 | 0.25 | 0.001041 | 0.0053445 |
| Th2 | *IL5RA; IL5RA;* | cg14749590 | 5′UTR; 5′UTR; 5′UTR; 5′UT | 3 | 0.08 | 0.4049314 | 0.56869602 | −0.01 | 0.877213 | 0.9513442 |
| Th2 | *IL5RA; IL5RA; IL5RA* | cg15621546 | Body; Body; 3′UTR; Body; B | 3 | −0.04 | 0.6731893 | 0.74050819 | 0.11 | 0.304844 | 0.5102828 |
| Th2 | *IL5RA; IL5RA; IL5RA; I* | cg23032421 | 5′UTR; 5′UTR; 1stExon; 5′ | 3 | 0.56 | 0.0005214 | 0.00365004 | 0.58 | 7.72E−12 | 2.97E−10 |
| Th2 | *IL5RA; IL5RA; IL5RA; I* | cg25381017 | 5′UTR; 5′UTR; 1stExon; 5′ | 3 | −0.01 | 0.935511 | 0.94782034 | 0.09 | 0.200767 | 0.4178127 |
| Th2 | *IL9* | cg12575771 | TSS200 | 5 | 0.09 | 0.1886998 | 0.38236536 | 0.05 | 0.434885 | 0.6318142 |
| Th2 | *IL9* | cg15966954 | TSS200 | 5 | 0.06 | 0.2703527 | 0.45254684 | 0.01 | 0.800807 | 0.9203309 |
| Th2 | *IL9* | cg25317724 | TSS1500 | 5 | 0.02 | 0.8250935 | 0.87030415 | 0.17 | 0.165157 | 0.3647101 |
| Th2 | *JAK1* | cg00153395 | Body | 1 | −0.22 | 5.77E−06 | 5.55E−05 | −0.38 | 6.29E−10 | 1.10E−08 |
| Th2 | *JAK1* | cg00420347 | 5′UTR | 1 | 0.08 | 0.4405439 | 0.59512074 | 0.04 | 0.621645 | 0.7977783 |
| Th2 | *JAK1* | cg07453451 | TSS1500 | 1 | 0.1 | 0.2131396 | 0.41029378 | −0.03 | 0.650362 | 0.8209482 |
| Th2 | *JAK1* | cg07798602 | 5′UTR | 1 | 0.07 | 0.2564974 | 0.43993838 | 0.14 | 0.014258 | 0.0522791 |
| Th2 | *JAK1* | cg09765463 | 5′UTR | 1 | 0.12 | 0.0699143 | 0.17944679 | 0.03 | 0.682918 | 0.8459211 |
| Th2 | *JAK1* | cg12075498 | 5′UTR | 1 | 0.17 | 0.4026188 | 0.56869602 | −0.07 | 0.711309 | 0.8557939 |
| Th2 | *JAK1* | cg12444684 | 5′UTR | 1 | −0.16 | 0.0292174 | 0.10713028 | −0.11 | 0.054605 | 0.1501644 |
| Th2 | *JAK1* | cg16250812 | 3′UTR | 1 | −0.13 | 0.2337164 | 0.42848008 | −0.1 | 0.381585 | 0.565986 |
| Th2 | *JAK1* | cg18227442 | 5′UTR | 1 | −0.02 | 0.8686265 | 0.89178989 | 0.12 | 0.225413 | 0.4347503 |
| Th2 | *JAK1* | cg25020373 | TSS200 | 1 | −0.08 | 0.3753413 | 0.56669173 | −0.28 | 0.00058 | 0.0031906 |
| Th2 | *JAK1* | cg26315985 | 5′UTR | 1 | −0.27 | 0.0134263 | 0.0552918 | −0.15 | 0.062399 | 0.1656797 |
| Th2 | *JAK3* | cg01089639 | TSS1500 | 19 | 0.13 | 0.5002163 | 0.64194422 | 0.1 | 0.590173 | 0.7702261 |
| Th2 | *JAK3* | cg02285920 | TSS200 | 19 | −0.24 | 0.2315016 | 0.42848008 | −0.27 | 0.049289 | 0.1445445 |
| Th2 | *JAK3* | cg06655414 | Body | 19 | 0.34 | 0.0393449 | 0.1262316 | 0.1 | 0.479733 | 0.6716262 |
| Th2 | *JAK3* | cg11085762 | Body | 19 | −0.15 | 0.4841302 | 0.63567769 | 0.19 | 0.285036 | 0.5062199 |
| Th2 | *JAK3* | cg12138330 | Body | 19 | 0.11 | 0.2415693 | 0.43257763 | 0.09 | 0.186053 | 0.397946 |
| Th2 | *JAK3* | cg21988119 | TSS200 | 19 | 0.23 | 0.1727747 | 0.35955821 | −0.02 | 0.8667 | 0.9513442 |
| Th2 | *JAK3* | cg25623545 | Body | 19 | 0.05 | 0.5472988 | 0.64833863 | −0.25 | 0.008182 | 0.0350365 |
| Th2 | *STAT6* | cg03848267 | TSS1500 | 12 | −0.16 | 0.1127107 | 0.26776539 | 0 | 0.989366 | 0.995332 |
| Th2 | *STAT6* | cg12693595 | 5′UTR | 12 | 0.16 | 0.011702 | 0.05300307 | 0.06 | 0.339706 | 0.5358969 |
| Th2 | *STAT6* | cg20779414 | TSS1500 | 12 | 0.14 | 0.1472224 | 0.33341538 | 0 | 0.995332 | 0.995332 |
| Th2 | *STAT6* | cg25157914 | TSS1500 | 12 | −0.05 | 0.5229052 | 0.64833863 | −0.15 | 0.035507 | 0.1139195 |
| Th2 | *STAT6; STAT6* | cg01063813 | 1stExon; 5′UTR | 12 | −0.12 | 0.2836308 | 0.46467177 | 0.07 | 0.541676 | 0.7191218 |
| Th17 | *IL17A* | cg05884768 | 3′UTR | 6 | −0.21 | 0.0458467 | 0.15494237 | −0.07 | 0.408062 | 0.609219 |
| Th17 | *IL17A* | cg27168844 | Body | 6 | −0.14 | 0.3060133 | 0.54113894 | 0.04 | 0.735042 | 0.8969358 |
| Th17 | *IL17B* | cg01202296 | TSS200 | 5 | 0.08 | 0.1341358 | 0.33853312 | 0.07 | 0.201829 | 0.4278772 |
| Th17 | *IL17B* | cg01579636 | TSS200 | 5 | 0.21 | 0.001967 | 0.01389986 | 0.13 | 0.010722 | 0.0473544 |
| Th17 | *IL17B* | cg03876697 | TSS1500 | 5 | 0.05 | 0.4055255 | 0.63543399 | 0.07 | 0.339351 | 0.5368834 |
| Th17 | *IL17B* | cg05860978 | TSS200 | 5 | −0.07 | 0.276372 | 0.50509363 | −0.12 | 0.014007 | 0.0593882 |
| Th17 | *IL17B* | cg18683707 | Body | 5 | 0.05 | 0.4871783 | 0.68854528 | 0 | 0.986701 | 0.9867006 |
| Th17 | *IL17B* | cg19424122 | Body | 5 | 0.09 | 0.2066261 | 0.42119943 | 0.04 | 0.494891 | 0.7086658 |
| Th17 | *IL17B* | cg26599373 | TSS1500 | 5 | 0 | 0.9519739 | 0.98015515 | 0.03 | 0.642322 | 0.8203148 |
| Th17 | *IL17C* | cg07794885 | TSS1500 | 16 | 0.15 | 0.0929388 | 0.25535887 | 0.02 | 0.834505 | 0.9370868 |
| Th17 | *IL17C* | cg08155347 | TSS200 | 16 | −0.21 | 0.0119514 | 0.05758385 | −0.12 | 0.321288 | 0.5368834 |
| Th17 | *IL17C* | cg21593409 | Body | 16 | 0.11 | 0.1718175 | 0.39121441 | 0.02 | 0.830913 | 0.9370868 |
| Th17 | *IL17C* | cg26686608 | Body | 16 | 0.14 | 0.0003268 | 0.00346387 | 0.14 | 0.009142 | 0.0421322 |
| Th17 | *IL17C; IL17C* | cg27132152 | 1stExon; 5′UTR | 16 | 0.18 | 0.0090531 | 0.04949836 | 0.23 | 0.003722 | 0.0197269 |
| Th17 | *IL17D* | cg02792322 | Body | 13 | −0.05 | 0.5531151 | 0.71651755 | −0.17 | 0.006062 | 0.0306002 |
| Th17 | *IL17D* | cg04906043 | Body | 13 | 0 | 0.9254632 | 0.98015515 | 0.05 | 0.5371 | 0.7206662 |
| Th17 | *IL17D* | cg05130518 | Body | 13 | 0.1 | 0.1484809 | 0.36602275 | 0.12 | 0.211531 | 0.4285184 |
| Th17 | *IL17D* | cg08195842 | 5′UTR | 13 | 0.05 | 0.2470599 | 0.46764902 | 0.01 | 0.788624 | 0.9370868 |
| Th17 | *IL17D* | cg08371706 | Body | 13 | 0.07 | 0.1822926 | 0.39121441 | 0.12 | 0.070083 | 0.1954939 |
| Th17 | *IL17D* | cg09985351 | Body | 13 | 0.2 | 0.0468176 | 0.15494237 | 0.21 | 0.067112 | 0.1922675 |
| Th17 | *IL17D* | cg12475590 | Body | 13 | 0.1 | 0.4653469 | 0.67559516 | −0.27 | 0.002361 | 0.0166836 |
| Th17 | *IL17D* | cg13303573 | Body | 13 | −0.1 | 0.1818016 | 0.39121441 | 0 | 0.971005 | 0.9802522 |
| Th17 | *IL17D* | cg14133014 | Body | 13 | −0.06 | 0.332272 | 0.568078 | 0.06 | 0.293106 | 0.5242378 |
| Th17 | *IL17D* | cg16851046 | Body | 13 | 0.12 | 0.4716419 | 0.67559516 | 0.17 | 0.301736 | 0.5242378 |
| Th17 | *IL17D* | cg23969506 | Body | 13 | −0.06 | 0.3680585 | 0.60959687 | 0.01 | 0.923767 | 0.9694978 |
| Th17 | *IL17D* | cg25189429 | 5′UTR | 13 | 0 | 0.9840862 | 0.98598505 | 0.12 | 0.167864 | 0.3631343 |
| Th17 | *IL17D; IL17D* | cg26790132 | 1stExon; 5′UTR | 13 | −0.05 | 0.4352476 | 0.65908916 | −0.06 | 0.337766 | 0.5368834 |
| Th17 | *IL17F* | cg01158657 | TSS200 | 6 | 0.05 | 0.7957088 | 0.90693686 | 0.13 | 0.362371 | 0.5648725 |
| Th17 | *IL17F* | cg03912011 | TSS1500 | 6 | 0.13 | 0.1125613 | 0.29828741 | 0.02 | 0.772224 | 0.9301786 |
| Th17 | *IL17F* | cg06280032 | Body | 6 | 0.03 | 0.5817404 | 0.73410097 | −0.06 | 0.419646 | 0.6178129 |
| Th17 | *IL17F* | cg11570484 | Body | 6 | 0.12 | 0.4465167 | 0.66663054 | −0.09 | 0.5081 | 0.7086658 |
| Th17 | *IL17F* | cg13540639 | TSS1500 | 6 | −0.01 | 0.9496511 | 0.98015515 | −0.14 | 0.117144 | 0.2822105 |
| Th17 | *IL17RA* | cg01085328 | Body | 22 | 0.17 | 0.0109181 | 0.05511022 | 0.02 | 0.665665 | 0.8400054 |
| Th17 | *IL17RA* | cg01760983 | Body | 22 | −0.65 | 0.0001149 | 0.00135309 | −0.24 | 0.03927 | 0.1300817 |
| Th17 | *IL17RA* | cg02866761 | TSS200 | 22 | −0.16 | 0.0011822 | 0.00895069 | −0.08 | 0.152525 | 0.3439931 |
| Th17 | *IL17RA* | cg13595439 | TSS1500 | 22 | 0.13 | 0.2137709 | 0.4259945 | 0.15 | 0.015053 | 0.0613682 |
| Th17 | *IL17RA* | cg15502903 | Body | 22 | −0.09 | 0.2295697 | 0.44244348 | −0.19 | 0.000176 | 0.0020731 |
| Th17 | *IL17RA* | cg16389078 | Body | 22 | −0.52 | 2.72E−08 | 7.20E−07 | −0.36 | 0.000573 | 0.0050596 |
| Th17 | *IL17RA* | cg19901866 | TSS1500 | 22 | 0.24 | 0.0322329 | 0.11781688 | −0.13 | 0.227698 | 0.4469631 |
| Th17 | *IL17RA* | cg21717745 | TSS1500 | 22 | −0.1 | 0.1315815 | 0.33853312 | −0.15 | 0.061053 | 0.1849043 |
| Th17 | *IL17RC; IL17RC;* | cg00657581 | Body; Body; | 3 | 0.13 | 0.3063051 | 0.54113894 | 0.02 | 0.85951 | 0.9419356 |
| Th17 | *IL17RC; IL17RC;* | cg16366604 | Body; Body; | 3 | 0.04 | 0.6589822 | 0.79377406 | 0.05 | 0.506678 | 0.7086658 |
| Th17 | *IL17RD* | cg00743540 | Body | 3 | 0.04 | 0.5207444 | 0.7076783 | 0.19 | 0.001817 | 0.0137605 |
| Th17 | *IL17RD* | cg00770158 | Body | 3 | −0.09 | 0.0289843 | 0.10972614 | −0.01 | 0.811319 | 0.9370868 |
| Th17 | *IL17RD* | cg01534707 | Body | 3 | 0 | 0.985985 | 0.98598505 | 0.03 | 0.839842 | 0.9370868 |
| Th17 | *IL17RD* | cg01797381 | 3′UTR | 3 | −0.29 | 0.0045176 | 0.02660364 | −0.32 | 0.000418 | 0.004029 |
| Th17 | *IL17RD* | cg03435901 | 3′UTR | 3 | −0.48 | 5.82E−05 | 0.00077143 | −0.4 | 0.003378 | 0.0197269 |
| Th17 | *IL17RD* | cg05138949 | Body | 3 | −0.05 | 0.3932062 | 0.63151306 | 0.13 | 0.099579 | 0.262977 |
| Th17 | *IL17RD* | cg09429700 | Body | 3 | −0.03 | 0.6465449 | 0.78774437 | 0.11 | 0.003631 | 0.0197269 |
| Th17 | *IL17RD* | cg09614565 | TSS200 | 3 | −0.27 | 0.0789876 | 0.23257461 | 0.06 | 0.605304 | 0.7893923 |
| Th17 | *IL17RD* | cg10882522 | TSS1500 | 3 | −0.28 | 0.0010847 | 0.00884428 | −0.18 | 0.0186 | 0.073022 |
| Th17 | *IL17RD* | cg11096815 | TSS200 | 3 | −0.07 | 0.5542872 | 0.71651755 | 0.06 | 0.529226 | 0.7206662 |
| Th17 | *IL17RD* | cg16763270 | Body | 3 | −0.08 | 0.2170161 | 0.4259945 | 0.1 | 0.158631 | 0.3503107 |
| Th17 | *IL17RD* | cg18745629 | Body | 3 | 0.07 | 0.3466505 | 0.58325324 | 0.08 | 0.214259 | 0.4285184 |
| Th17 | *IL17RD* | cg20075859 | Body | 3 | 0.12 | 0.0939528 | 0.25535887 | 0.15 | 0.147421 | 0.3397094 |
| Th17 | *IL17RD* | cg23727321 | TSS200 | 3 | −0.16 | 0.1613546 | 0.38871792 | 0.02 | 0.933058 | 0.969648 |
| Th17 | *IL17RD; IL17RD* | cg15854067 | 1stExon; 5′UTR | 3 | 0.04 | 0.5902811 | 0.73611526 | 0.01 | 0.835053 | 0.9370868 |
| Th17 | *IL17RE; IL17RC; IL17R* | cg05845178 | 3′UTR; TSS1500; 3′UTR; 3′ | 3 | 0.06 | 0.5445724 | 0.71651755 | −0.08 | 0.295505 | 0.5242378 |
| Th17 | *IL17RE; IL17RE;* | cg02968508 | Body; Body | 3 | −0.11 | 0.0247403 | 0.1008643 | −0.05 | 0.375301 | 0.5683131 |
| Th17 | *IL17RE; IL17RE; IL17R* | cg05253480 | 1stExon; 5′UTR; 1stExon; | 3 | 0.2 | 0.0165116 | 0.07000898 | 0.22 | 0.08253 | 0.2243127 |
| Th17 | *IL17RE; IL17RE; IL* | cg06619959 | Body; Body; | 3 | −0.16 | 0.002543 | 0.01648424 | −0.1 | 0.002943 | 0.0194964 |
| Th17 | *IL17RE; IL17RE;* | cg07832674 | TSS1500; TSS1500; | 3 | −0.19 | 0.5074666 | 0.7076783 | −0.04 | 0.839796 | 0.9370868 |
| Th17 | *IL17RE; IL17RE; IL17R* | cg15095327 | 1stExon; 5′UTR; 1stExon; | 3 | 0.13 | 0.0428671 | 0.15146363 | 0.19 | 0.031088 | 0.1155382 |
| Th17 | *IL17RE; IL17RE;* | cg18738581 | TSS200; TSS1500; | 3 | 0.03 | 0.7319314 | 0.85257938 | −0.09 | 0.304292 | 0.5242378 |
| Th17 | *IL17RE; IL17RE; IL17R* | cg19148440 | Body; Body; Body | 3 | 0.27 | 0.3124768 | 0.54299253 | 0.08 | 0.726782 | 0.8969358 |
| Th17 | *IL17REL* | cg00090674 | 5′UTR | 22 | −0.02 | 0.8876453 | 0.98015515 | 0.23 | 0.000216 | 0.002289 |
| Th17 | *IL17REL* | cg00692279 | 5′UTR | 22 | 0.12 | 0.0004281 | 0.00412487 | 0.13 | 0.006574 | 0.0316766 |
| Th17 | *IL17REL* | cg01522296 | TSS1500 | 22 | 0.01 | 0.8964235 | 0.98015515 | 0.02 | 0.736164 | 0.8969358 |
| Th17 | *IL17REL* | cg04485799 | Body | 22 | 0.14 | 0.0258157 | 0.10135055 | 0.13 | 0.038925 | 0.1300817 |
| Th17 | *IL17REL* | cg05736785 | Body | 22 | −0.01 | 0.9524149 | 0.98015515 | 0.08 | 0.370135 | 0.5683131 |
| Th17 | *IL17REL* | cg07312716 | Body | 22 | 0.03 | 0.5636351 | 0.71982316 | −0.04 | 0.531723 | 0.7206662 |
| Th17 | *IL17REL* | cg12009803 | 5′UTR | 22 | 0.11 | 0.0133152 | 0.0613656 | 0.08 | 0.105917 | 0.262977 |
| Th17 | *IL17REL* | cg13563334 | 5′UTR | 22 | 0.05 | 0.1953966 | 0.40611852 | 0.15 | 1.95E−05 | 0.0003454 |
| Th17 | *IL17REL* | cg16744710 | 5′UTR | 22 | −0.01 | 0.9444823 | 0.98015515 | 0.04 | 0.610662 | 0.7893923 |
| Th17 | *IL17REL* | cg19796981 | Body | 22 | 0.04 | 0.6799486 | 0.80323611 | 0.1 | 0.213507 | 0.4285184 |
| Th17 | *IL17REL* | cg26206185 | TSS200 | 22 | 0.55 | 2.30E−05 | 0.00034794 | 0.22 | 0.106679 | 0.262977 |
| Th17 | *IL17REL* | cg27068297 | TSS1500 | 22 | −0.14 | 0.0026437 | 0.01648424 | −0.1 | 0.064612 | 0.1902471 |
| Th17 | *IL21* | cg14340103 | TSS1500 | 4 | −0.09 | 0.3910598 | 0.63151306 | −0.01 | 0.913884 | 0.9687165 |
| Th17 | *IL21* | cg18051361 | 1stExon | 4 | 0.1 | 0.08204 | 0.23503359 | 0.04 | 0.443901 | 0.6445687 |
| Th17 | *IL21; IL21* | cg00136405 | 5′UTR; 1stExon | 4 | −0.16 | 0.0140485 | 0.0620474 | −0.16 | 0.003601 | 0.0197269 |
| Th17 | *IL21R* | cg02656594 | TSS1500 | 16 | −0.53 | 2.86E−14 | 3.04E−12 | −0.52 | 2.89E−15 | 3.06E−13 |
| Th17 | *IL21R; IL21R* | cg00050618 | TSS200; 5′UTR | 16 | 0.26 | 0.0006364 | 0.00562145 | 0.22 | 3.05E−06 | 6.46E−05 |
| Th17 | *IL21R; IL21R* | cg02513379 | TSS200; 5′UTR | 16 | −0.03 | 0.6436564 | 0.78774437 | −0.11 | 0.103566 | 0.262977 |
| Th17 | *IL21R; IL21R* | cg04059988 | TSS1500; TSS200 | 16 | 0.05 | 0.4091992 | 0.63543399 | −0.01 | 0.887588 | 0.9600443 |
| Th17 | *IL21R; IL21R* | cg05814654 | TSS1500; TSS1500 | 16 | −0.86 | 5.46E−06 | 0.00011569 | −0.82 | 0.000101 | 0.0015308 |
| Th17 | *IL21R; IL21R* | cg16454902 | TSS200; 5′UTR | 16 | −0.1 | 0.1845351 | 0.39121441 | −0.13 | 0.059211 | 0.1845991 |
| Th17 | *IL21R; IL21R; IL21R* | cg02983090 | TSS1500; 5′UTR; 5′UTR | 16 | −0.72 | 9.87E−06 | 0.00017444 | −0.73 | 2.14E−07 | 7.52E−06 |
| Th17 | *IL21R; IL21R; IL21R* | cg03093786 | TSS1500; 1stExon; 5′UTR | 16 | −0.01 | 0.9259592 | 0.98015515 | −0.13 | 0.30663 | 0.5242378 |
| Th17 | *IL21R; IL21R; IL21R* | cg08282819 | TSS1500; 5′UTR; 5′UTR | 16 | −1.07 | 5.33E−09 | 1.88E−07 | −0.96 | 4.29E−14 | 2.27E−12 |
| Th17 | *IL21R; IL21R; IL21R* | cg19423311 | TSS1500; 1stExon; 5′UTR | 16 | −0.48 | 0.0482518 | 0.15494237 | −0.41 | 0.035241 | 0.1245198 |
| Th17 | *IL21R; IL21R; IL21R* | cg26505691 | 3′UTR; 3′UTR; 3′UTR | 16 | −0.21 | 0.178173 | 0.39121441 | −0.1 | 0.569041 | 0.7539794 |
| Th17 | *IL21R; IL21R; IL21R* | cg27027151 | 3′UTR; 3′UTR; 3′UTR | 16 | −0.47 | 4.78E−11 | 2.53E−09 | −0.33 | 2.84E−07 | 7.52E−06 |
| Th17 | *IL22* | cg02543402 | TSS200 | 12 | 0.16 | 0.1677058 | 0.39121441 | −0.14 | 0.259563 | 0.4852425 |
| Th17 | *IL22* | cg03694077 | 3′UTR | 12 | 0 | 0.9702341 | 0.98598505 | −0.11 | 0.259795 | 0.4852425 |
| Th17 | *IL22* | cg13195526 | TSS1500 | 12 | −0.01 | 0.8832061 | 0.98015515 | 0 | 0.9539 | 0.9785793 |
| Th17 | *IL22* | cg13851647 | TSS200 | 12 | 0.05 | 0.6819929 | 0.80323611 | −0.22 | 0.046456 | 0.1492213 |
| Th17 | *IL22* | cg14588682 | TSS200 | 12 | −0.11 | 0.4136316 | 0.63543399 | 0.01 | 0.960116 | 0.9785793 |
| Th17 | *IL22* | cg27018240 | TSS200 | 12 | 0.06 | 0.515513 | 0.7076783 | 0.01 | 0.913815 | 0.9687165 |
| Th17 | *IL22* | cg27518047 | TSS1500 | 12 | −0.01 | 0.9110087 | 0.98015515 | −0.01 | 0.86196 | 0.9419356 |
| Th17 | *IL22; IL22* | cg11520493 | 1stExon; 5′UTR | 12 | −0.14 | 0.2738805 | 0.50509363 | −0.17 | 0.136668 | 0.3219291 |
| Th17 | *IL22RA1* | cg02072322 | TSS1500 | 1 | 0.08 | 0.4566466 | 0.67228531 | 0.12 | 0.328504 | 0.5368834 |
| Th17 | *IL22RA1* | cg09152089 | 1stExon | 1 | 0.16 | 0.0673492 | 0.20397191 | 0.18 | 0.03161 | 0.1155382 |
| Th17 | *IL22RA1* | cg11651446 | 3′UTR | 1 | 0.02 | 0.7507795 | 0.86502858 | 0.17 | 0.001762 | 0.0137605 |
| Th17 | *IL22RA1* | cg21293216 | TSS200 | 1 | 0.28 | 0.0093393 | 0.04949836 | 0.4 | 0.000155 | 0.0020521 |
| Th17 | *IL22RA2; IL22RA2; IL2* | cg00415333 | 5′UTR; 5′UTR; 5′UTR | 6 | 0.14 | 0.0496985 | 0.15494237 | 0.1 | 0.33686 | 0.5368834 |
| Th17 | *IL22RA2; IL22RA2; IL2* | cg23564241 | 1stExon; 5′UTR; 5′UTR; 1s | 6 | −0.03 | 0.5507045 | 0.71651755 | 0.08 | 0.260932 | 0.4852425 |
| Treg | *CTLA4; CTLA4* | cg14288266 | 3′UTR; 3′UTR | 2 | 0.09 | 0.5568359 | 0.58052654 | −0.08 | 0.501366 | 0.6267069 |
| Treg | *CTLA4; CTLA4* | cg24077172 | TSS1500; TSS1500 | 2 | −0.08 | 0.4633968 | 0.57924597 | −0.13 | 0.335646 | 0.4794945 |
| Treg | *CTLA4; CTLA4* | cg26091609 | Body; Body | 2 | 0.04 | 0.5805265 | 0.58052654 | −0.17 | 0.052108 | 0.1042157 |
| Treg | *FOXP3; FOXP3* | cg01905377 | TSS1500; TSS1500 | 26 | −1.01 | 2.19E−08 | 1.09E−07 | −1.28 | 7.46E−11 | 7.46E−10 |
| Treg | *FOXP3; FOXP3* | cg02033323 | 5′UTR; 5′UTR | 26 | −0.17 | 0.0572203 | 0.12076323 | −0.16 | 0.205028 | 0.3417127 |
| Treg | *FOXP3; FOXP3* | cg04920616 | TSS200; TSS200 | 26 | 0.14 | 0.218366 | 0.31195139 | 0.21 | 0.040058 | 0.100145 |
| Treg | *FOXP3; FOXP3* | cg06767008 | Body; Body | 26 | 0.42 | 7.46E−12 | 7.46E−11 | 0.37 | 2.22E−05 | 0.0001111 |
| Treg | *FOXP3; FOXP3* | cg15614573 | TSS1500; TSS1500 | 26 | −0.41 | 0.0333016 | 0.11100529 | −0.81 | 0.001281 | 0.0042697 |
| Treg | *FOXP3; FOXP3* | cg16350494 | TSS1500; TSS1500 | 26 | −0.12 | 0.157382 | 0.26230332 | −0.05 | 0.692998 | 0.6929983 |
| Treg | *FOXP3; FOXP3; FOXP3; F* | cg10858077 | 1stExon; 5′UTR; 1stExon; | 26 | 0.1 | 0.0603816 | 0.12076323 | −0.03 | 0.605006 | 0.6722287 |

Table 5. Percentages of CpGs showed significant change in DNA−M from age 18 to first and second halves of pregnancy in randomly selected groups of CpGs from whole genome, genes more expressed in blood and from whole genome without CpGs situated in the intergenic region (10 sets in each group).

| Total Number of CpGs (Randoms) | **First half of pregnancy** | | | | | | | | | | | **Second half of pregnancy** | | | | | | | | | | | | | | | | |  |  |
| --- | --- | --- | --- | --- | --- | --- | --- | --- | --- | --- | --- | --- | --- | --- | --- | --- | --- | --- | --- | --- | --- | --- | --- | --- | --- | --- | --- | --- | --- | --- |
|  | **Random subsets of CpGs from whole genome** | | | | | | | | | | | | | | | | | | | | | | | | | | | | | |
|  | Number of Significant CpGs | | | Percentage | | | Number of CpGs survived after FDR test | | Percentage | | | | | Number of Significant CpGs | | Percentage | | | | | Number of CpGs survived after FDR test | | | | Percentage | | | | |  |
| R1 (348) | 81 | 23.3 | | | | 42 | | 12.1 | | | | | 82 | | 23.6 | | | | | 41 | | | | 11.8 | | | |  |  |  |
| R2 (348) | 77 | 22.1 | | | | 41 | | 11. 8 | | | | | 85 | | 24.49 | | | | | 48 | | | | 13.8 | | | |  |  |  |
| R3 (348) | 84 | 24.1 | | | | 47 | | 13.5 | | | | | 91 | | 26.19 | | | | | 53 | | | | 15.2 | | | |  |  |  |
| R4 (348) | 89 | 25.6 | | | | 57 | | 16.4 | | | | | 90 | | 25.9 | | | | | 51 | | | | 14.7 | | | |  |  |  |
| R5 (348) | 84 | 24.1 | | | | 43 | | 12.4 | | | | | 84 | | 24.19 | | | | | 53 | | | | 15.2 | | | |  |  |  |
| R6 (348) | 90 | 25.9 | | | | 59 | | 17.0 | | | | | 86 | | 24.79 | | | | | 53 | | | | 15.2 | | | |  |  |  |
| R7 (348) | 85 | 24.4 | | | | 38 | | 10.9 | | | | | 83 | | 23.9 | | | | | 47 | | | | 13.5 | | | |  |  |  |
| R8 (348) | 89 | 25.6 | | | | 48 | | 13.8 | | | | | 103 | | 29.6 | | | | | 65 | | | | 18.7 | | | |  |  |  |
| R9 (348) | 81 | 23.3 | | | | 47 | | 13.5 | | | | | 79 | | 22.7 | | | | | 43 | | | | 12.4 | | | |  |  |  |
| R10 (348) | 79 | 22.7 | | | | 43 | | 12.4 | | | | | 92 | | 26.4 | | | | | 64 | | | | 18.4 | | | |  |  |  |
| Total (3480) | 839 | 24.1 | | | | 465 | | 13.4 | | | | | 875 | | 25.1 | | | | | 518 | | | | 14.9 | | | |  |  |  |
|  | **Random subsets of CpGs from genes expressed in CD4+ cells** | | | | | | | | | | | | | | | | | | | | | | | | | | | | | |
| R1 (348) | 83 | 23.9 | | | 56 | | | | | 11.89 | | | 81 | | | | 23.3 | | 43 | | | | 8.27 | | | |  |  |  |  |
| R2 (348) | 91 | 26.1 | | | 54 | | | | | 11.46 | | | 96 | | | | 27.6 | | 58 | | | | 11.2 | | | |  |  |  |  |
| R3 (348) | 84 | 24.1 | | | 55 | | | | | 11.68 | | | 90 | | | | 25.9 | | 62 | | | | 11.9 | | | |  |  |  |  |
| R4 (348) | 76 | 21.8 | | | 35 | | | | | 7.43 | | | 85 | | | | 24.49 | | 50 | | | | 9.6 | | | |  |  |  |  |
| R5 (348) | 82 | 23.6 | | | 46 | | | | | 9.77 | | | 97 | | | | 27.9 | | 52 | | | | 10.0 | | | |  |  |  |  |
| R6 (348) | 92 | 26.4 | | | 42 | | | | | 8.92 | | | 98 | | | | 28.2 | | 58 | | | | 11.15 | | | |  |  |  |  |
| R7 (348) | 84 | 24.1 | | | 51 | | | | | 10.83 | | | 100 | | | | 28.7 | | 46 | | | | 8.9 | | | |  |  |  |  |
| R8 (348) | 86 | 24.7 | | | 50 | | | | | 10.62 | | | 98 | | | | 28.7 | | 62 | | | | 11.9 | | | |  |  |  |  |
| R9 (348) | 78 | 22.4 | | | 48 | | | | | 10.19 | | | 81 | | | | 23.3 | | 50 | | | | 9.6 | | | |  |  |  |  |
| R10 (348) | 77 | 22.1 | | | 34 | | | | | 7.22 | | | 72 | | | | 20.7 | | 39 | | | | 7.5 | | | |  |  |  |  |
| Total (3480) | 833 | 23.9 | | | 471 | | | | | 13.5 | | | 898 | | | | 25.8 | | 520 | | | | 14.9 | | | |  |  |  |  |
|  | **Random groups of CpGs (whole genome) excluding CpGs situated in intergenic region** | | | | | | | | | | | | | | | | | | | | | | | | | | | | | |
| R1 (348) | 87 | | 25 | | 56 | | | | | 16.1 | 93 | | | | | 26.7 | | 55 | | | | 15.8 | | | |  |  |  |  |  |
| R2 (348) | 87 | | 25 | | 51 | | | | | 14.7 | 89 | | | | | 25.6 | | 47 | | | | 13.5 | | | |  |  |  |  |  |
| R3 (348) | 86 | | 24.7 | | 53 | | | | | 15.2 | 88 | | | | | 25.3 | | 57 | | | | 16.43 | | | |  |  |  |  |  |
| R4 (348) | 89 | | 25.6 | | 50 | | | | | 14.4 | 95 | | | | | 27.3 | | 58 | | | | 16.7 | | | |  |  |  |  |  |
| R5 (348) | 92 | | 26.4 | | 59 | | | | | 17.0 | 101 | | | | | 29.0 | | 69 | | | | 19.8 | | | |  |  |  |  |  |
| R6 (348) | 88 | | 25.3 | | 57 | | | | | 16.4 | 98 | | | | | 28.2 | | 68 | | | | 19.5 | | | |  |  |  |  |  |
| R7 (348) | 76 | | 21.8 | | 29 | | | | | 8.3 | 93 | | | | | 26.7 | | 55 | | | | 15.8 | | | |  |  |  |  |  |
| R8 (348) | 81 | | 23.3 | | 46 | | | | | 13.2 | 82 | | | | | 23.6 | | 49 | | | | 14.1 | | | |  |  |  |  |  |
| R9 (348) | 83 | | 23.9 | | 43 | | | | | 12.4 | 79 | | | | | 22.7 | | 34 | | | | 9.8 | | | |  |  |  |  |  |
| R10 (348) | 89 | | 25.6 | | 55 | | | | | 15.8 | 85 | | | | | 24.4 | | 58 | | | | 16.7 | | | |  |  |  |  |  |
| Total (3480) | 858 | | 24.7 | | 499 | | | | | 14.3 | 903 | | | | | 25.9 | | 550 | | | | 15.8 | | | |  |  |  |  |  |

Table 6. Distribution of adjacent CpG sites (+/− 1500 base-pairs) with significant changes in methylation from age 18 (non-pregnant state) to first and second halves of pregnancy.

| Direction of change in DNA-M of CpGs from non-pregnant state to pregnancy | **Th1 (155 CpGs)** | | **Th2 (77 CpGs)** | | | | **Th17 (106 CpGs)** | | | | **Treg (10 CpGs)** | | | |  |  |
| --- | --- | --- | --- | --- | --- | --- | --- | --- | --- | --- | --- | --- | --- | --- | --- | --- |
|  | CpGs not spatially clustered *n* (%) | Adjacent CpGs & changing in same direction *n* (%) | CpGs not spatially clustered n (%) | | Adjacent CpGs & changing in same direction *n* (%) | | CpGs not spatially clustered *n* (%) | | Adjacent CpGs & changing in same direction *n* (%) | | CpGs not spatially clustered *n* (%) | | Adjacent CpGs & changing in same direction *n* (%) | |  |  |
| **First half of pregnancy** | | | | | | | | | | | | | | | | |
| Increase in methylation | 5 (3.2) | 3 (1.9) | | 1 (1.3) | | 9 (11.7) ^a^ | | 2 (1.9) | | 9 (8.5) | | 0 (0) | | 0 (0) | |  |
| Decrease in methylation | 10 (6.5) | 15 (9.7) | | 7 (9.1) | | 2 (2.6) | | 9 (8.5) ^a^ | | 6 (5.7) | | 0 (0) | | 2 (20) | |  |
| No significant change | 38 (24.5) | 84 (54.2) | | 34 (44.2) | | 24 (31.2) | | 26 (24.5) | | 54 (50.9) | | 5 (50) | | 3 (30) | |  |
| **Second half of pregnancy** | | | | | | | | | | | | | | | | |
| Increase in methylation | 8 (5.2) | 10 (6.5) | 2 (2.6) | | 6 (7.8) | | 3 (2.8) | | 8 (7.5) | | 0 (0) | | 1 (10) | |  |  |
| Decrease in methylation | 10 (6.5) | 18 (11.6) | 8 (10.4) ^a^ | | 1 (1.3) | | 8 (7.5) ^a^ | | 4 (3.8) | | 0 (0) | | 2 (20) ^a^ | |  |  |
| No significant change | 35 (22.6) | 74 (47.7) | 32 (41.6) | | 28 (36.4) | | 26 (24.5) | | 57 (53.8) | | 5 (50) | | 2 (20) | |  |  |

^a^ Indicates a significant difference (*p* ≤ 0.05) compared to the distribution in non-significant changes during pregnancy.

**Table 7**. Comparing the number of significant CpGs in the group of 20 participants assessed in both first and second halves of pregnancy with 39 participants in first half and 35 participants in second half of pregnancy. Non-pregnant state (age 18) is the reference for all change.

| Pathways (Total number of CpGs) | **First Half of pregnancy** | | | **Second Half of pregnancy** | | |
| --- | --- | --- | --- | --- | --- | --- |
|  | Total number of significant CpGs (%) | | | Total number of significant CpGs (%) | | |
|  | Group of 20 participants | Group of 39 participants | Chi-square  *p*-Value | Group of 20 participants | Group of 35 participants | Chi-square *p*-Value |
| Th1 (155) | 37 (23.9) | 43 (27.7) | 0.964 | 37 (23.9) | 56 (36.1) | 0.818 |
| Th2 (77) | 20 (26.0) | 27 (35.1) |  | 20 (26.0) | 26 (33.8) |  |
| Th17 (106) | 30 (28.3) | 34 (32.1) |  | 25 (23.6) | 33 (31.1) |  |
| Treg (10) | 3 (30.0) | 3 (30.0) |  | 5 (50.0) | 4 (40.0) |  |
